# Mismatch Responses to Speech Contrasts in Preschoolers with and without Developmental Language Disorder

**DOI:** 10.3390/brainsci14010042

**Published:** 2023-12-31

**Authors:** Ana Campos, Jyrki Tuomainen, Outi Tuomainen

**Affiliations:** 1 UCL Ear Institute, University College London, London WC1E 6BT, UK; 2 Department of Speech, Hearing and Phonetic Sciences, University College London, London WC1N 1PF, UK; jyrtuoma@gmail.com; 3 Carrera de Fonoaudiología, Universidad San Sebastián, Lota 2465, Santiago 7510602, Chile; 4 Department of Linguistics, University of Potsdam, 14469 Potsdam, Germany; tuomainen@uni-potsdam.de

**Keywords:** DLD, multifeature paradigm, mismatch response, ERP time–frequency analysis

## Abstract

This study compared cortical responses to speech in preschoolers with typical language development (TLD) and with Developmental Language Disorder (DLD). We investigated whether top-down language effects modulate speech perception in young children in an adult-like manner. We compared cortical mismatch responses (MMRs) during the passive perception of speech contrasts in three groups of participants: preschoolers with TLD (n = 11), preschoolers with DLD (n = 16), and adults (n = 20). We also measured children’s phonological skills and investigated whether they are associated with the cortical discrimination of phonemic changes involving different linguistic complexities. The results indicated top-down language effects in adults, with enhanced cortical discrimination of lexical stimuli but not of non-words. In preschoolers, the TLD and DLD groups did not differ in the MMR measures, and no top-down effects were detected. Moreover, we found no association between MMRs and phonological skills, even though the DLD group’s phonological skills were significantly lower. Our findings suggest that top-down language modulations in speech discrimination may not be present during early childhood, and that children with DLD may not exhibit cortical speech perception deficits. The lack of association between phonological and MMR measures indicates that further research is needed to understand the link between language skills and cortical activity in preschoolers.

## 1. Introduction

Between the ages of three and six years, children can understand spoken language effortlessly. However, preschoolers do not perceive speech as efficiently as adults do, and there may also be differences between children of the same age who have different language skills, for example, children with typical language development (TLD) versus those with Developmental Language Disorder (DLD). DLD, previously referred to as Specific Language Impairment (SLI), affects around 7% of children and is characterised by a range of language deficits, especially in morphosyntactic and phonological processing, without an identifiable medical cause [1]. The underlying causes of DLD are still largely unknown, but many studies suggest that children with language difficulties process speech differently from their typically developing peers [2,3].

However, the neural mechanisms underlying speech processing in young children are not well understood. Despite plenty of behavioural and brain imaging research, we do not know yet what cortical patterns of speech perception are typical at preschool age or whether these patterns vary between children and adults or between children with TLD and DLD [4]. Understanding this is important because early childhood is a period of significant language growth, and spoken language perception is critical for children’s later communication and literacy development. It is also during early childhood when many children with language disorders are first diagnosed, suggesting that some language symptoms could become more apparent at this age. Therefore, identifying neural patterns associated with typical and atypical speech processing in children could aid the development of objective clinical measures, fostering the earlier detection of and intervention in language disorders. Importantly, this would require determining adult reference values first, representing the neurotypical, mature cortical speech-processing patterns against which to compare children’s responses. 

As for the role of speech perception deficits in DLD, different hypotheses have pointed out that specific problems with processing speech sounds could be an underlying marker of this disorder [5,6]. However, there is no clarity about the nature of these difficulties. Some early theories of DLD state that low-level speech perception deficits are a hallmark of DLD. For example, difficulties in detecting rapid acoustic changes within speech sounds or extracting distinctive acoustic cues from the speech stream could lead to unstable phonological representations, which then manifest as phonological deficits, such as difficulties in repeating non-words (see [7] for a review). Other accounts have proposed that speech perception deficits in DLD could be a consequence and not a cause of language difficulties [8], where less efficient speech processing in DLD derives from limited language skills, and not vice versa. For example, poorer phonological processing skills in DLD than in TLD may affect children’s ability to detect subtle acoustic changes in speech sounds but not in other sound contrasts [9]. Notably, several studies have failed to detect any evidence of atypical speech processing in DLD [10], suggesting that either these deficits do not exist or they cannot be detected with the current methods. So far, neurobiological findings are contradictory, making it difficult to identify any neural markers of speech perception deficits in DLD.

An advantageous method to investigate the brain processing of complex, rapidly changing acoustic signals, such as speech, is the electroencephalogram (EEG) because of its high temporal resolution. From the EEG, it is possible to extract event-related potential (ERP) components, such as the Mismatch Negativity (MMN), which has been extensively used across a wide range of ages and populations [11,12], and the less-studied Late Discriminative Negativity (LDN). Unlike early auditory detection responses, such as the P50/P1/N1 components, the MMN and LDN reflect cortical sound discrimination, a process that occurs later in the brain, at fixed latencies (time-locked), after a change in auditory stimulation. In adults, the auditory MMN is a negative deflection of about 0.5–5 µV that occurs 100–250 ms after a discriminable change in any acoustic feature [12]. The LDN appears later (250–400 ms) and seems to be more pronounced in children than in adults [13] and for auditory contrasts involving speech rather than nonspeech sounds [14], for which some studies consider it a signature of sound processing complexity [15]. In infants and young children, the MMN and LDN may present an immature form, so mismatch response (MMR) is a generic term to refer to these responses deviating from the adult-like pattern [16]. Like the adult MMN/LDN, the MMR reflects the brain’s sensitivity to physical and abstract changes (“deviants”) in a sequence of regular sounds (“standards”) in many acoustic contrasts, including speech. The MMR/MMN/LDN can be elicited during unattended listening, making it a valuable measure when behavioural responses are not possible or are less reliable, as in young children or clinical groups [12]. 

In speech perception research, the MMR/MMN/LDN is used as a pre-attentive discrimination index of general auditory processing elicited by low-level, physical changes (i.e., in pitch, duration, or intensity) but also of speech-specific processing elicited by changes in higher-level, abstract linguistic representations (e.g., phonemic categories, lexical status, or word classes). Thus, the MMR/MMN/LDN not only depends on the bottom-up (afferent) processing of the speech acoustic features but is also influenced in a top-down manner by psycholinguistic factors, such as the listener’s knowledge of phonological categories and structures, as well as the words’ grammatical function, distributional frequency, and meaning [17]. Evidence from adults indicates that language-specific top-down mechanisms selectively facilitate the processing of speech [18], developing gradually during childhood from around the age of 7 years [19]. However, there is little evidence about how top-down modulations of speech perception operate at different stages of language development or whether they are impaired in children with language difficulties. Considering several studies indicating reduced or slower MMR/MMN/LDN in DLD (see [20] for a review), it is possible that the MMR/MMN/LDN response could serve as a neural marker of speech-specific difficulties in DLD.

One potential factor contributing to this gap in knowledge about the underlying nature and cause of DLD is the methodological complexity of conducting MMR research in young children (especially in clinical populations) and comparing groups of children and adults [21]. Children are less able to tolerate long, repetitive testing sessions and may become fussy, introducing movement-related artefacts in the EEG. There are also neuroanatomical differences between adults and children (e.g., in head size, skull and cortical thickness, cortical fibre density) that complicate a direct comparison of their ERP responses [22]. In general, adults’ ERPs are intrinsically smaller and more consistent in timing than those of children, which are larger and much more variable [23]. Thus, when studying speech perception development across broad age ranges, it would be appropriate to complement conventional time-domain measures (e.g., amplitude and latency) with time–frequency measures (e.g., changes in spectral power or phase coherence over time). This would allow the measurement of important cortical oscillatory activity that is not consistently time-locked to the stimulus and would be otherwise lost in ERP averaging [24]. In the next sections, we will review what is known about the speech-elicited MMR/MMN/LDN patterns in young children with and without DLD and in adults, both from studies using conventional ERP measures and from those using time–frequency analysis.

### 1.1. MMR/MMN/LDN in Speech Perception Development

Multiple studies have shown that the MMR changes from birth to adulthood, reflecting auditory and brain maturation [16,25,26]. Throughout development, the MMR can present a different polarity, latency, amplitude, and scalp distribution than the adult MMN/LDN. In infants and young children, the MMR polarity is usually reversed towards positive values [27], with some studies reporting positive MMRs until the age of 6–7 years [28,29]. Others, however, report negative, MMN-like responses much earlier, i.e., in the first six months of life [30]. MMR scalp localisation is more broadly distributed in children than the adult MMN/LDN, which is more focalised and shows the maximum amplitude at the frontocentral electrodes (e.g., Fz, Cz) [31,32]. 

Regarding temporal patterns, the MMR latency correlates negatively with age during infancy and childhood, with delayed and longer responses in young children than in adults [16,23]. In infants between 7 and 11 months, MMRs have been reported with a latency of 250–500 ms [33]. In three year old children, there is evidence of the MMR peaking between 120 and 400 ms, whereas, for 5–8-year-olds, peak latencies occurred between 190 and 270 ms, a more adult-like range [28]. Shafer et al. [34] observed similar latencies using narrower age bands, with later and longer MMRs in 4–5 than in 6–7-year-old children, whereas Bishop et al. [13] reported similar latencies in children (age: 7–12 years) and adolescents (age: 13–16 years), in both cases slower than in adults. On the contrary, some studies indicate adult-like latencies much earlier, for example, in children between 6 and 13 years [35]. The MMR amplitude, however, seems not to follow a linear trajectory but a U-shaped curve during development [25]. Adult-like amplitudes are often observed in infants, with significantly smaller responses in early childhood (until around 7–8 years) until late childhood (12–13 years), followed by an increase in amplitudes until late adolescence (16–18 years). Paquette et al. [27] reported smaller MMRs in children (age: 3–13 years) than in adults and in younger (age 3–7 years) than in older children (age 8–13 years). Bishop et al. [13] reported significant age-related increases in mean amplitude for the MMN time window from childhood (children between 7 and 12 years) to adolescence (13–16 years) and from adolescence to adulthood (35–56 years) with amplitude decreases in the LDN time window for the same age groups. Other studies, however, report that the MMR amplitude is slightly smaller in infants but as large as, or larger than, in children above 6 years of age and in adults [36]. On the contrary, some studies indicate an amplitude reduction from early childhood to adult age. A longitudinal study by Chen, Tsao, and Liu [37] in Mandarin speakers detected an MMR amplitude reduction from preschool (mean = 3.40 years) to school age (mean = 8.57 years) to adulthood (mean = 22.4 years). In sum, despite the variability in the findings, the MMR seems to become more stable with age, showing a greater amplitude, shorter latency, and more localised negativity until finally reaching the fully mature pattern during early adulthood (see [38] for a review). 

Another aspect to consider is that, like the adult MMN/LDN, children’s MMR patterns reflect the acoustic and linguistic content (e.g., phonological, lexical, semantic information) in the speech input. Thus, there are differences in the MMR elicited by speech versus other nonspeech sounds [13,27,35] and for speech involving different linguistic contrasts. For example, some studies have reported larger MMRs to speech (syllables) than for acoustically matched nonspeech sounds in infants [38] and 6-year-old children [39]. However, in adults, other studies indicate the opposite: a smaller MMN for speech stimuli than for their acoustically matched nonspeech counterparts [40]. For speech stimuli, MMRs show different age-related trajectories for different linguistic features, such as native versus non-native phonemic contrasts [41], word versus non-words, or even distinctions between different word classes [42]. MMRs to non-native phonemic contrasts have been reported at the age of 7 months disappearing by the age of 11 months, whereas the MMR to native phonemes becomes more robust during the same period [33]. For native phonemes, Finnish children at age three years show MMN-like responses in the 300–400 ms range for vowel contrasts [43], whereas French-speaking children of the same age show adult-like MMNs, peaking at 270 ms for syllables with initial consonant contrasts (/bag/versus/da/) [27]. 

MMRs are also modulated by the linguistic context [42], for example, by the type of syllable or word in which speech sounds are presented. David et al. [14] investigated the discrimination of syllables with different phonological complexities, reporting smaller MMN-like but larger LDN-like responses in children (age: 6–10 years) than in adults for more complex syllables, but no difference between adults and children for less complex ones. Other studies report MMR enhancement when the deviant syllable occurs in a word compared to when it occurs in isolation or in a non-word, which has been linked to top-down lexical or semantic modulations. In 3-year-old children, Strotseva-Feinschmidt et al. [44] reported the effects of the word’s lexical frequency on the presence or absence of MMRs to two German function words (articles *der*/*den*). They found that the high-frequency article *der* elicited an MMN-like and an LDN-like response. In contrast, the low-frequency *den* elicited only an LDN, suggesting easier processing of higher-frequency words. In adults, there is evidence of larger MMNs for words than for non-words, indicating an enhancement of the MMN amplitude by the lexical-semantic content of the stimulus [17,45].

In children with DLD, cortical speech processing and its MMR signatures are less well understood, especially when compared with other neurodevelopmental disorders, such as developmental dyslexia or autism [46]. Overall, compared to TD children, children with DLD show poorer and slower cortical discrimination of speech sounds, resulting in smaller MMN amplitudes, delayed latencies, atypical scalp distributions, and less left-hemisphere lateralisation than TLD children (for a review, see [20]). Furthermore, other MMN findings suggest that children with DLD have difficulties in processing the acoustic features of speech and in detecting phonemic contrasts in their native language [21,29,47]. However, other studies have reported no differences in MMR between children with DLD and typically developing children (see [21] for a review). For example, a magnetoencephalography (MEG) study by Pihko et al. [48] in children between 5 and 7 years compared MMRs to syllables with changing vowels or consonants and detected no differences between children with DLD and controls. Similarly, Bishop, Hardiman, and Barry [49] compared the discrimination of phonemes with “small” and “large” differences between the standard and the deviants (e.g., standard/ba/vs. small deviant/da/and large deviant/bi/) in children and teenagers with DLD and TLD aged 7–16 years. They reported no group differences for the MMN and LDN amplitude for large deviants, although the LDN was reduced in the DLD group for small deviants. 

With regard to the debate concerning the underlying causes of DLD, several studies suggest a connection between the MMR/MMN/LDN and language skills, both in children with TLD and DLD. For example, studies in infants aged 7.5 to 24 months have reported a positive correlation between the MMR amplitude for native phonemic contrasts and behavioural phoneme discrimination measures [33,50]. Furthermore, Linnavalli et al. [51] demonstrated that children (age 5–6 years) with better phoneme discrimination performance showed larger MMRs than those with poorer behavioural results. Similarly, a study in preschoolers (mean age: 5.6 years) by Norton et al. [52] found significantly larger MMRs in the late time window (300–500 ms) for/ba/-/da/contrasts in children with typical phonological awareness (PA) skills than in those with low PA skills. In 2-month-old infants at risk for DLD, Friederich et al. [53] found delayed MMRs to vowel deviants with different durations. Overall, the findings indicate that the MMR amplitude correlates with language abilities in children with DLD, with weaker or slower MMRs associated with poorer language outcomes (see [20] for a review). More specifically, the evidence indicates a reduced amplitude, particularly over the left scalp areas, and delayed latency in infants and children at familial risk for language deficits or with a DLD diagnosis.

The MMR may also predict children’s receptive language skills at later ages [5]. Guttorm et al. [54] found that the MMN measured in infants 1–6 days after birth with and without a risk of developmental dyslexia predicted pre-reading language skills at the age of five years. Specifically, positive MMRs in the right hemisphere were associated with lower phonological, rapid naming, and letter knowledge skills. However, other studies show no relationship between the MMR and behavioural language measures [55], especially at the individual level [10].

In sum, the evidence indicates that MMR patterns in children change as a function of age, which suggests the ongoing maturation of cortical speech processing until later childhood. In addition to this, there is also evidence of different developmental rates for different linguistic features at the cortex. Furthermore, the associations between MMRs to speech sounds and later language skills suggest that the MMR could be a valuable tool for predicting language outcomes in children with DLD.

### 1.2. MMR Time–Frequency Analysis and Speech Perception Development

Although time–frequency analysis (TF) is less popular than conventional ERP analysis in the MMN research field, it may help further our understanding of how speech perception develops in early childhood and in clinical populations in particular. TF analysis measures the non-stimulus-locked neural oscillatory activity that is abundant in children and cancelled out by the ERP technique [56]. Moreover, TF analysis increases the ERP signal-to-noise ratio (SNR) [57], which is an advantage when dealing with noisy data (as is often the case in children’s EEG), making it more dependable than time-domain measures for MMR/MMN/LDN identification [55].

In adults, studies using TF analysis of the MMN response have found increased neural synchronisation in the theta frequency range (4 to 7–8 Hz) for deviants vs. standard sounds, for example, during the discrimination of sound duration contrasts. Fuentemilla et al. [58] found greater theta inter-trial phase coherence (ITPC) at the temporal and frontal electrode sites and event-related spectral perturbation (ERSP) for deviant (1000 Hz, 25 ms duration) than for standard (1000 Hz, 75 ms) tones at the frontal electrodes. Similarly, two studies in adults by Hsiao and colleagues [59,60] showed larger theta-phase-locking values (PLVs) and spectral power for duration deviants (1000 Hz, 50 ms duration) than for standards (1000 Hz, 100 ms duration). Bishop at al. [13] reported no changes in ERSP power but a significant increase in theta ITPC during MMN generation, which they considered an index of event-related oscillatory phase resetting. Although these findings indicate a role for increased theta ITPC in auditory deviance detection and the generation of the MMN [55], most of them were elicited by nonspeech stimuli, so it is unclear whether they can be generalised to speech sound processing.

In paediatric research, the few studies using TF analysis of the MMN/MMR suggest a relationship between increased stimulus-induced phase synchronisation and developmental changes in auditory perception. A longitudinal study by Bishop et al. [61] showed that between the ages of 7 and 11 years, there was an increase in theta ITPC for tone deviants in the frontal–central but not in the temporal regions, indicating the greater involvement of areas related to top-down modulations as children grow up. Studies by Müller et al. [62] and Poulsen et al. [63] reported that greater theta phase synchrony for deviants than for standards was present in children, and it increased from childhood to early adolescence, indicating more efficient sound detection. Bishop et al. [13] observed age-related increases in theta phase synchrony for deviant sounds, with the largest ITPC for adults (35–56 years) and larger ITPC for adolescents (13–16 years) than for children (7–12 years). Together, these findings indicate that the maturation of the MMN neural substrates is accompanied by age-related increases in oscillatory synchronisation, mainly in the theta range and frontal cortical regions, suggesting more consistent neural responses and more involvement of areas involved in top-down processing.

Notably, some renowned infant studies using speech stimuli suggest that age-related increases in spectral power and ITPC in the delta, theta, and gamma bands between the ages of 6 and 12 months may reflect selective enhancement and perceptual narrowing for native-language phonemes [64,65]. Moreover, in adults, theta synchronisation is thought to play a critical role in syllabic segmentation [66]. However, there is little research on the developmental trajectories of different theta-band measures. So far, there is little information about how theta brain activity is related to the linguistic content of the speech stimuli or to the language skills in children with TLD and DLD.

Cortical oscillatory dynamics have been far less investigated in DLD than in other neurodevelopmental disorders, such as developmental dyslexia or autism spectrum disorder (for a review, see [56]). However, there is some evidence indicating that atypical oscillatory activity may underlie language disorders [46]. Bishop et al. [49] compared the cortical discrimination of tones and speech sounds in children (7 to 16 years old) with DLD and TLD, measuring low-frequency-band synchronisation in the MMN/LDN intervals. Even though they found no between-group differences in the MMN, the TLD (but not the DLD) group had a significant drop in power in the LDN versus the MMN interval for the low-frequency bands (delta, theta, and alpha). The authors suggested that this lack of event-related desynchronisation in the DLD group after the MMN indicates an inability to disengage neural activity after initially “normal” auditory discrimination responses [49]. Two other studies by Heim et al. [67,68] examined oscillatory dynamics during rapid auditory processing of tone pairs in children between 6 and 9 years with and without language disorders. In the language-impaired group, they found that atypical early processing (45–75 ms) significantly reduced the gamma (29–52 Hz range) amplitude and phase-locking values. The authors interpreted these findings as evidence of altered oscillatory timing in language-impaired children when processing rapid sequences of tones. Again, they used nonspeech or simple speech stimuli, making it hard to draw conclusions about how the linguistic content of the stimuli might modulate the brain responses.

In conclusion, the MMR is a valuable tool for investigating the neural mechanisms underlying speech processing in children. In typically developing children, the MMR has an identifiable developmental trajectory, and it indicates sensitivity to speech contrasts, positively correlating with later language development. In children with DLD, the MMR has shown reduced amplitudes and delayed latencies, suggesting difficulties in processing the acoustic features of speech sounds. However, only a few studies have exploited the advantages of MMR time–frequency measures to characterise children’s responses to speech sounds and conduct comparisons between adults and children.

### 1.3. The Current Study

The purpose of this study was to compare the cortical discrimination of speech with linguistic content of varying complexity in participants with different language skills: preschoolers with TLD (typical developmental status), preschoolers with DLD (atypical developmental status), and adults. Although some previous studies have compared similar groups, only a few have tested young children, and most have included relatively broad age ranges. Also, previous studies in children have usually focused on specific phonological contrasts or investigated word versus non-word processing, but, to our knowledge, none have examined how multiple linguistic levels (phonology, semantic content, and grammatical class of a word) modulate the MMR in the same study.

Therefore, we investigated whether, during early childhood, cortical responses to speech are modulated by top-down language skills, as we previously confirmed they do in adults, and whether children with DLD show atypical MMR patterns to different types of linguistic stimuli. To compare top-down modulations in the MMR, we included linguistic stimuli of varying complexity (native versus non-native phonemes, words versus non-words, content versus function words) presented in the same experiment with a multifeature paradigm. This paradigm alternates several deviants against one standard, increasing the number of contrasts, without losing statistical power, which reduces the EEG testing time considerably (see, e.g., [69,70]). To account for previous methodological limitations, we complemented conventional ERP measures (latency and amplitude) with time–frequency indices (ERSP and ITPC). Because the previous literature has linked phonological awareness deficits to speech-processing difficulties in DLD, we also assessed children’s phonological skills and examined whether they were associated with the MMR measures.

Based on previous findings, we hypothesised that an MMR would be present in children for all speech contrasts. However, we expected that the MMR patterns would vary between the TLD and DLD groups and between children and adults because of group-level differences in language skills and the interaction of top-down modulations with the linguistic content of the stimuli. Specifically, we predicted that cortical responses would be (1) more immature (e.g., positive instead of negative polarity of MMRs), (2) less robust (e.g., smaller amplitude and longer latency), and (3) less synchronised (e.g., reduced ERSP and ITPC) in the DLD group than in TLD children and in children than in adults. According to our earlier paradigm validation study in adults (Campos et al., in prep), we also expected that the TLD group, but not the DLD group, would show more robust and synchronised MMRs to stimuli with lexical content (words) than those involving only phonological contrasts (non-words). Finally, in both groups of children, we expected MMR measures to correlate with phonological awareness test performance. 

## 2. Materials and Methods

### 2.1. Participants

Twenty-nine monolingual Spanish-speaking children between the ages of 4.9 and 5.7 years were invited to the study. However, two children were excluded from the study due to non-compliance with the EEG procedure, leaving the final sample at 27 children. Participants were recruited in Santiago, Chile, and divided into two groups according to their language status: a group with a previous diagnosis of expressive–receptive Developmental Language Disorder (DLD, n = 16, 6 female, M age 5.2 years, SD = 0.33, range 4.9–5.7 years) and a group of age-matched controls with typical language development (TLD, n = 11, 7 female, M age 5.2 years, SD = 0.23, range 4.10–5.6 years). To control (as much as possible) for socioeconomic factors, all children were recruited from the same public preschool in Santiago, Chile, ensuring that they all lived within the geographical school catchment area, and that their parents/carers had completed at least secondary education (in Chile, mandatory until the age of 18 years).

Children in the DLD group were diagnosed at least one year before this study by a Speech and Language Therapist (SLT) as part of the initial assessment for the preschool admission of children who are at risk of language difficulties or whose parents are concerned about their language development The diagnosis was based on the Chilean legislation for Language Special Preschools and is requested by a paediatrician, child neurologist, or psychiatrist whenever a language disorder is suspected. The SLT assessment includes a full parental interview and medical history, functional orofacial and hearing check, speech sound production screening, and three standardised language tests that assess language comprehension and production skills at the morphosyntactic, lexical, and semantic levels. Although the school assessment records were not available due to data protection restrictions, all children in the DLD group had a diagnosis of expressive–receptive DLD variant and met the following criteria: (i) being affected by language difficulties that significantly impair their day-to-day communication, (ii) exhibiting significantly poor performance on the three aforementioned language tests (scores 2 SD below the age-expected norm), and (iii) not being affected by other concomitant neurodevelopmental disorders, health conditions, or environmental factors that explain the language deficit. 

For the TLD control group, age-matched children with no significant medical history were selected from the mainstream division of the same preschool as the DLD group. For both groups, all children who met the criteria were invited via a letter to their parents, and those whose parents returned the signed consent form and completed a background questionnaire underwent the study screening process. Only those children who passed a hearing screening (otoscopy and play audiometry at 500, 1000, 2000, and 4000 Hz) and were able to complete a nonverbal reasoning task (Block Design subtest of the Wechsler Intelligence Scale for Children, WISC [71]) were recruited for the study. Importantly, the tests used for DLD diagnosis were not used again as variables in this study to avoid the drawbacks of testing children too often with the same tools (e.g., boredom or item memorisation). 

In addition, data from 20 native Chilean Spanish-speaking adults (12 female, age, M age 34.2 years, range 24.9–44.11 years, SD = 4.8) from a previous validation study conducted at University College London, Infant and Child Language Lab were included for age-related comparisons (Campos et al., in prep). Neurotypical adults were invited to participate after confirming that they met the following inclusion criteria: (i) pure tone average (PTA) air-conduction thresholds ≤20 dB for both ears at octave frequencies from 500 to 4000 Hz or a threshold of ≤25 dB at any given frequency from 250 to 8000 Hz, (ii) performing no more than 1 SD below the normative mean (M = 50, SD = 5) on the Block Design test of the Wechsler Abbreviated Scale of Intelligence (WASI) [72], a standardised measure of nonverbal IQ, and (iii) being a native Chilean Spanish speaker. Before recruitment, adults completed an online screening questionnaire about their medical and language history. Those adults who met any of these criteria were excluded from the study: (i) a previous or current diagnosis of DLD or any other neurodevelopmental disorder, hearing loss, or any neurological conditions; (ii) not using Spanish as their main language (currently or in the past). All adults had English as their second language with different levels of proficiency, but none considered themselves native English speakers, and only two reported speaking in English before the age of 2 years, although not consistently. Table 1 presents the screening information for both groups of children and for the reference adult group. 

This study was conducted in accordance with the Declaration of Helsinki and approved by the Research Ethics Committees of University College London (UCL) and Universidad de Chile. In all cases, participants or their parents/guardians received an information sheet plus a verbal explanation of the study, completed a developmental questionnaire, and signed a consent form before the screening phase. Children provided verbal assent before testing started. Parents received GBP 10 for travel expenses, and children received a small age-appropriate gift.

### 2.2. Stimuli

#### 2.2.1. Phonological Awareness Task

Behavioural testing focused on children’s phonological awareness skills, as they were more directly associated with the speech cortical discrimination process of our interest and the stimulus contrasts in the ERP experiment (see Section 2.2.2). We assessed participants’ phonological skills using the syllabic subsection of the Phonological Awareness Assessment Test, PECFO (Prueba de Evaluacion de Conciencia Fonologica) [73]. This test has been normed and standardised for Chilean children between 4 and 7.11 years, although it is not used in DLD diagnosis, thus lacking unwanted re-testing effects. The syllabic subsection of this test measures six different phonological awareness (PA) skills: syllable segmentation, initial syllable recognition, rhyme recognition, initial syllable deletion, rhyme deletion, and syllable inversion. Each task consisted of five items, with one point assigned for each correct item and a maximum score of 30 for the subsection.

#### 2.2.2. ERP Experiment

The stimulus set consisted of five CVC monosyllables created according to the Spanish language phonotactic rules: one standard (288 in total) and four deviants (72 stimuli for each type, 288 deviants in total) with a total of 576 stimuli. Stimuli were recorded by a female native Chilean Spanish speaker in an acoustically shielded booth at a 44.100 Hz sampling rate in stereo channels. During pre-processing, we cut each stimulus from the recorded string, defining its beginning/end to the nearest zero crossing. The stimulus duration ranged from 610 to 680 ms with a 15 ms ramp-on/off segment. The intensity of all stimuli was normalised to the root-mean-square (RMS) at 66.7 dB. 

The five stimuli are presented in Table 2. They consisted of one standard (St, a non-word with a native initial phoneme) and four deviants (D1 to D4) produced by changing the initial phoneme of the standard stimulus while keeping constant the vowel nucleus and the final consonant. These phonemic changes resulted in acoustic and phonological contrasts between the standard and deviant stimuli aiming to elicit the MMR but also involved different levels of linguistic processing: (i) phonological: native (D1) versus non-native (D2) phonemes in non-words (phonotactically allowed word forms without meaning); (ii) lexical: native non-words (D2) versus real words (D3 + D4); and (iii) semantic: function (D3) versus content words (D4). 

Stimuli were controlled as much as possible for acoustic and linguistic differences known to influence the MMR. According to Guardia [74], the initial phonemes were selected to maximise their similarity in terms of linguistic (e.g., syllable structure, word length/stress) and lexical factors (age of acquisition and oral frequency). Thus, the stimuli met the following criteria: (i) St and D2 are non-words with an initial native Spanish phoneme, (ii) D1 is a non-word with an initial phoneme that is non-native in Spanish, (iii) D3 is a function word in Chilean Spanish, (iv) D4 is a content word in Chilean Spanish, and (v) D3 and D4 are similar in their age of acquisition and oral frequency and are acquired before the age of 4.6 years, according to Spanish lexical databases by Alonso et al. [75] and Corral et al. [76].

As illustrated in Table 2, “fus” (a non-word in Spanish) was selected as the standard (St) stimulus. To create deviant 1 (D1), the initial phoneme of the St was changed into /∫/, but the vowel and final consonant were preserved (/u/ and /s/, respectively), resulting in the non-word /∫us/ (“shus”), which is non-native in Spanish. For deviant 2 (D2), the initial phoneme was changed into /x/, a native Spanish phoneme that produced the non-word /xus/ (“hus”). For deviant 3 (D3), the initial phoneme was /t/, resulting in the function word /tus/ (“tus”, meaning “yours”), and for deviant 4 (D4), the initial phoneme was /l/, producing the content (lexical) word /lus/ (“luz”, meaning “light”). Although a fricative onset consonant (in St, D1, and D2) and the /u/ vowel nucleus could make the stimulus less salient (because of greater noise and lower amplitude, respectively), only these CVC combinations met all the criteria for our experiment.

**Table 2:** Linguistic parameters for standard and deviant stimuli.

Type	Class	Initial Consonant	Vowel	Final Consonant	Age of Acquisition ^a^	Oral Frequency ^b^
St	Non-word	/f/ Native, labiodental, unvoiced fricative	/u/	/s/	--	--
D1	Non-word	/∫/ Non-native, postalveolar, unvoiced fricative	/u/	/s/	--	--
D2	Non-word	/x/ Native, velar, unvoiced fricative	/u/	/s/	--	--
D3	Function word (determiner)	/t/ Native, alveolar, unvoiced stop	/u/	/s/	4.24 ^a^	2.63 ^b^
D4	Content word (noun)	/l/ Native, alveolar, voiced, lateral	/u/	/s/	3.18 ^a^	2.53 ^b^

Note. St: standard; D1: deviant 1; D2: deviant 2; D3: deviant 3; D4: deviant 4. ^a^ Subjective AoA in years [75]. ^b^ Among the 100 most frequent words and monosyllables in Spanish [76].

### 2.3. Procedure 

To reduce the data collection time, we used a multifeature experiment, previously validated by our lab in a group of adults. Stimuli were delivered using MATLAB 2016a and a Fireface interface, presented free field at 60 dB via loudspeakers in front of the participants. 

**Figure 1:**
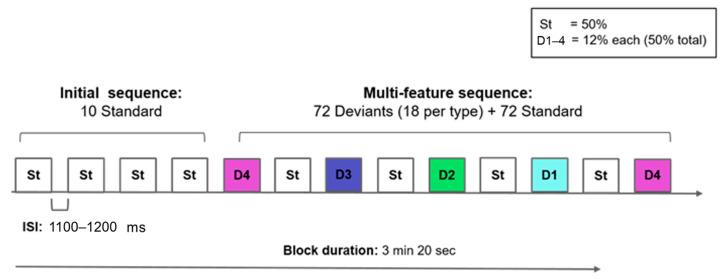
Structure of each experimental block for the multifeature experiment. Each experimental block consisted of an initial sequence of 10 standards (native non-words, in white squares) and a multifeature sequence, in which D1 (non-native non-words, in light blue), D2 (native non-words, in green), D3 (function words, in blue), and D4 (content words, in pink) were randomly alternated with the standard. Total number of stimuli per block = 154. ISI: inter-stimulus interval.

Stimuli were divided into four blocks of 3 min 20 seconds duration, with a total duration of around 15 min (including breaks). Each experimental block consisted of an initial habituation sequence of 10 standards, and a multifeature sequence of 144 stimuli, as displayed in Figure 1. In the multifeature sequence, the four deviants (12% of the trials each) were interspersed in a randomised order with the standard stimulus (50% of the trials). The inter-stimulus interval (ISI) was randomly jittered between 1100 and 1200 ms to avoid a rhythmic presentation.

Children were tested in three sessions, all conducted on separate days in Santiago, Chile. The first session took place at the children’s preschool after their parent/guardian signed the informed consent form and answered a developmental questionnaire. Children who provided verbal assent underwent a hearing screening consistent with otoscopy and play audiometry (pass/fail at 500–1000–2000–4000 Hz, 40 and 20 dB) and performed the Block Design task from the WASI [71]. The second and third sessions were conducted three months later to allow enough time to analyse the screening data and make logistic arrangements in the testing facilities at Neurosistemas Lab, Universidad de Chile. Here, the EEG was recorded in a sound-attenuated booth, with stereo stimuli presented free field at 70 dB through right and left loudspeakers at 90 cm at a 75-degree angle. During the experiment, no response was required, and children sat comfortably in an armchair with their parents next to them while watching a silent cartoon on a tablet screen that was placed at eye level in front of them at a distance of 100 cm. Participants were instructed that they would hear some sounds via the loudspeaker and not to attend to them. A research assistant supervised to ensure that children remained still, alert, and engaged with the cartoons. Each EEG session lasted around 15 min (with breaks when needed) plus a set-up time of 20 min. To avoid children’s exhaustion, the final session was held separately three weeks later and consisted of the phonological awareness task [72].

### 2.4. EEG Acquisition and Processing

Continuous EEG was recorded with a 32-channel Biosemi system at a 2048 Hz sampling rate. Electrodes were positioned according to the 10–20 electrode system, with offsets kept under 30 µV. Vertical and horizontal electro-oculograms were recorded in the right supraorbital area and right eye canthus, respectively. The EEG was preprocessed with EEGLab [77] and ERPLab [78]. EEG data were downsampled to 500 Hz and re-referenced offline to the full-head average. A high-pass IIR Butterworth filter (non-causal, zero-phase shift, 2nd order) with a cut-off of 0.1 Hz (roll-off 12 dB/octave attenuation, half amplitude -6dB, half-power -3dB) was applied to the continuous EEG to remove slow drifts [79]. An initial threshold of 350 µV was applied to remove data portions with excessively large artefacts. To retain as much data as possible, we visually inspected each dataset and removed noise-contaminated data portions and channels. Then, we performed ICA to remove blinks, eye movements, and other artefacts. After data cleaning, we interpolated the removed channels and re-referenced the data to the full head average. Then, separate pipelines were applied for the ERP and time–frequency analysis. 

For ERP analysis, epochs were defined from −200 to 800 ms, with baseline correction between −200 and 0 ms. In total, 619 epochs of 1000 ms duration were extracted per participant. Epochs with artefacts exceeding an absolute threshold of 200 µV were excluded. We quantified the EEG noise level as the percentage of epochs rejected per participant for each stimulus type and condition, with an individual maximum artefact rejection criterion of 35% of the trials per stimulus type and a minimum of 44 trials per deviant condition. All ERP statistical analyses were performed in the subtracted difference waveforms (DW, deviant minus standard) using Mass Univariate Analysis [80], with the peak-centred mean amplitude and peak latency as measures, calculated in two time windows: TW1 (100–250 ms) and TW2 (250–400 ms). Importantly, no participants were excluded from any of the groups, as all datasets were below the rejection threshold after data cleaning and artefact correction.

Time–frequency analysis was performed with Fieldtrip [81] in the parent waves for each standard and deviant type, following the previous literature (e.g., [82]). We used Morlet wavelets for the spectral decomposition of each trial into 19 log-spaced frequencies from 2 to 45 Hz. Morlet wavelet parameters were defined according to the previous literature (e.g., [57]), using 3 cycles at the lowest and 14 at the highest frequency (0.8-cycle increase) and a window length of 1670 ms. To avoid edge artefacts, non-overlapping epochs of 3000 ms duration were defined between −1000 and 2000 ms for each trial and then averaged across deviant types for each participant. Baseline correction was applied from −500 to −200 ms to avoid spectral leakage from the following epoch in the low frequencies. Running the decomposition trial by trial allowed us to obtain induced activity and avoid cancelling out responses that were not time-locked. As time–frequency analysis is sensitive to differences in the number of trials per condition [57], we found the participant with the minimum number of trials for a given condition (60 trials) and matched this number in all other participants and conditions by randomly selecting 60 trials from each participant’s pool. 

The brain’s oscillatory synchrony in a given frequency band was examined through two time–frequency measures: ERSP and ITPC. ERSP quantified how much energy the signal had for each frequency at each time point and was measured as the power change relative to the −500 to −200 baseline (in dB), averaged across conditions for each group [57]. ITPC was calculated as an index between 0 and 1 and reflected how consistently the oscillations reach the same point in the cycle across stimulus types. 

### 2.5. Statistical Analysis

This study considered a between-subject design for comparing speech processing under different language statuses: typically developing, atypically developing, and adult-like. The between-subject factor (IV) was different language status (operationalised as “Group”), with three levels: TLD children, DLD children, and adults. Our EEG dependent variable (DV) was cortical responses to speech, operationalised as ERP (peak latency, in ms, and mean amplitude, in µV) and time–frequency measures (ERSP, in dB power, and ITC, in a 0–1 range). At the behavioural level, the DV consisted of the phonological awareness scores for the syllabic awareness test subsection. 

Statistical analyses were conducted with MATLAB and SPSS v26-29. For the ERP analysis, we identified significant MMRs in the TLD and DLD groups using Mass Univariate Analysis (MUA [80]; successive point-by-point *t*-tests with FDR control [83]) in a broad time window between 100 and 500 ms. Then, we ran between-group comparisons of the peak-centred mean amplitude in the early (TW1, 100–250 ms) and late (TW2, 250–400 ms) windows separately using one-way ANOVA with Bonferroni correction and Tamhane’s post hoc tests if necessary. 

For TF analysis, we wanted to avoid bias when selecting the time windows for ERSP and ITPC. Thus, we determined regions of interest (ROIs) for the theta and alpha bands in a way that was blind to the stimulus type by averaging together the responses to all stimulus types for each group. We compared ERSP and ITPC between participant groups and used a separate mixed repeated-measures ANOVA for each ROI, with Bonferroni correction for multiple comparisons. Effect sizes were measured with eta-squared (η2) and partial eta-squared (ηp2), considering a large effect ≥ 0.14, a medium effect ≥ 0.06, and small effect ≥ 0.01; Cohen’s d (large effect = 0.8; medium effect = 0.5; small effect = 0.2); or epsilon-squared (ε2, with large effect > 0.36; medium effect > 0.04 and < 0.36; small effect < 0.04), as appropriate.

In terms of confound control, there were no differences between the groups of children in the screening variables age (U = 86.5, *p* = 0.960) and hearing level (PTA left ear: U = 86, *p* = 0.927; PTA right ear: U = 66, *p* = 0.230), but the DLD group showed significantly lower nonverbal scores than the control group (Block Design test: U = 44, *p* = 0.011), with a medium effect size (d = 0.5). However, nonverbal scores did not meet the required assumptions to be used as adequate covariates in the child groups: they were not normally distributed, and there was no linearity between them and any of the DVs. Thus, they were not used as covariates to avoid invalidating the results of any analysis including them as such (e.g., Analysis of Covariance, ANCOVA), but this difference in nonverbal skills was considered when interpreting our findings. Importantly, although the DLD group performed worse in the Block Design test, their scores were still within the normal range.

## 3. Results

### 3.1. Behavioural Results

After confirming data normality, independent-samples *t*-tests revealed that the DLD group (M = 14.57, SD = 4.67) showed significantly lower scores than the TLD group (M = 20.87, SD = 5.86) for the phonological awareness test (t(19) = 2.778, *p* = 0.012), with a large effect size, d = 1.23. As adults would likely show ceiling effects in children’s language tests because of their fully developed auditory, language, and cognitive skills, we did not compare adults and children in terms of phonological awareness skills. 

### 3.2. EEG Results

#### 3.2.1. ERP Analysis of MMRs

##### Characteristics of ERP Responses

**Figure 2:**
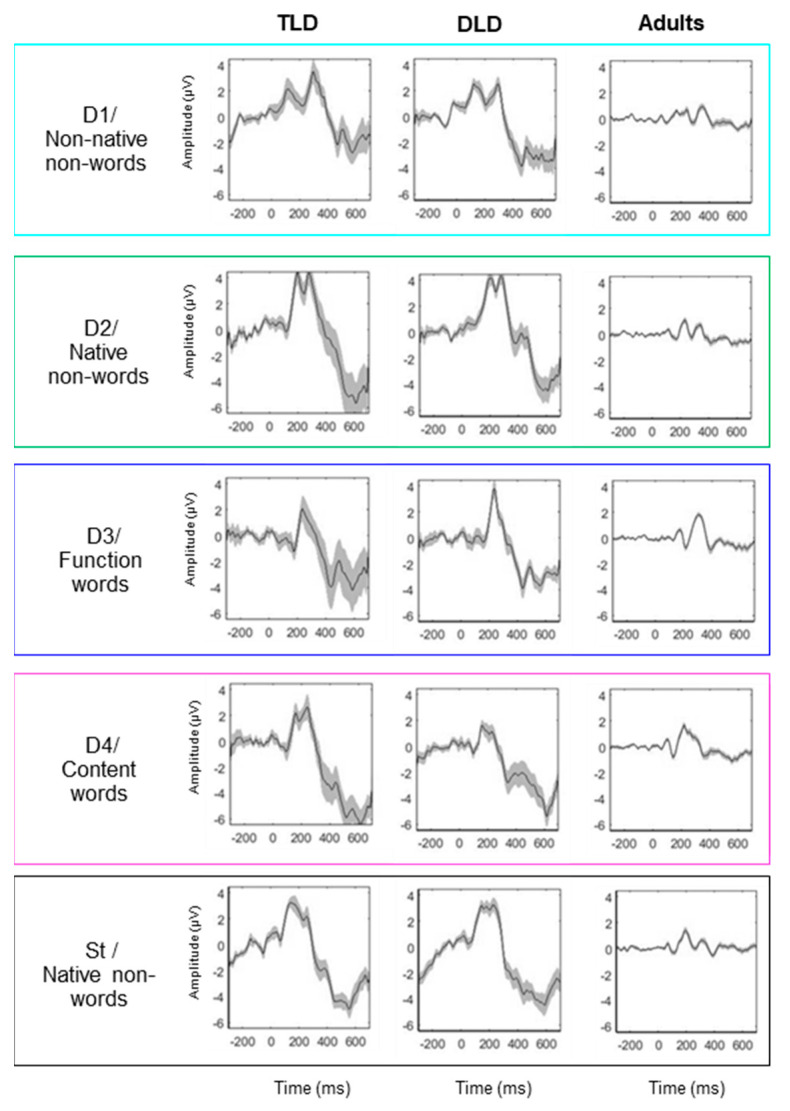
ERPs at Fz, grand average for all groups and stimulus types. Parent waveforms for the TLD (left column), DLD (middle column), and adult groups (right column). D1: non-native non-words (light blue box); D2: native non-words (green box); D3: function words (blue box); D4: content words (pink box); and St: native non-words (standards, black box). Central lines: mean ERPs; shaded areas: standard error (SE). Time 0 = stimulus onset.

Grand average ERP waveforms were computed at Fz for all stimulus types, consistent with the previous literature and with our validation study in adults that showed the maximum effect at this electrode in this experiment. Visual inspection of the grand average waveforms (see Figure 2) indicates the presence of obligatory auditory responses in all groups for all the stimulus types, with clear positive peaks around 200 and 300 ms and negative responses before 200 and after 400 ms. However, Figure 2 shows a considerably greater ERP amplitude and standard error in the DLD and TLD groups than in adults, with larger and more variable deflections in children. This indicates that ERP patterns are similar in both groups of children, regardless of their language status. On the contrary, there are differences between children and adults in terms of the magnitude, polarity, timing, and variability of the responses.

**Figure 3:**
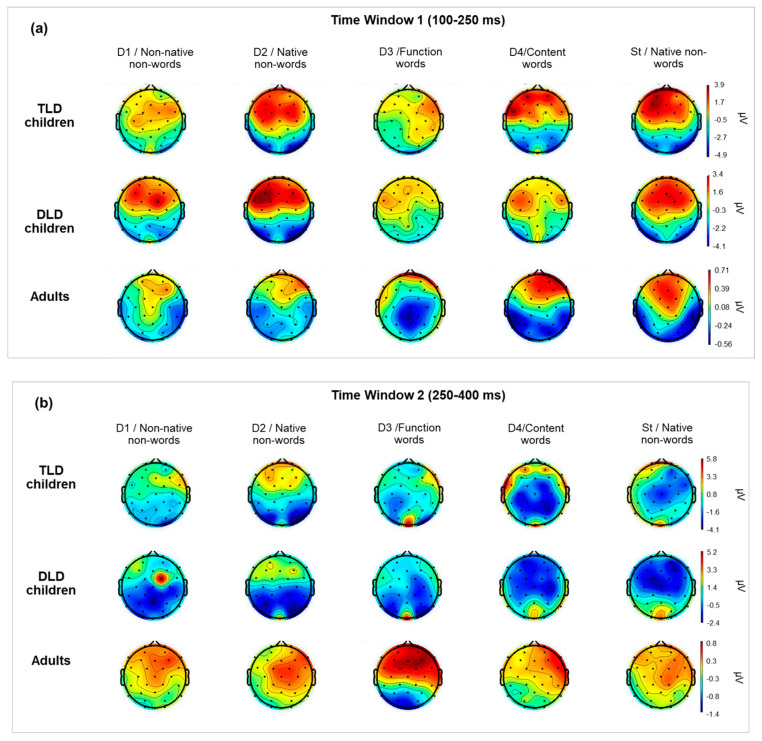
ERP scalp distribution for each stimulus type in all groups. Panel (**a**) TW1: 100–250 ms; Panel (**b**) TW2: 250–400 ms. In each panel, columns from left to right are as follows: D1: non-native non-words; D2: native non-words; D3: function words; D4: content words; St: standards, native non-words. Colour bar: µV.

Scalp distributions were computed for each stimulus type by averaging the ERP amplitudes across TW1 (100–250 ms) and TW2 (250–400 ms), as displayed in Figure 3a,b, respectively. In both time windows, responses in children had considerably larger magnitudes and showed different polarities and less focalised activation than in adults. In general, the scalp patterns for TW1 (Figure 3a) showed frontal–central positive and posterior negative polarities. The activation was similar in adults and children for the standard stimuli, but for the deviants, adults showed more localised frontal positivity and clearer temporal negativity than children. The responses in TLD and DLD children were broadly distributed and mostly of positive polarity in TW1 and negative polarity in TW2. The activation was similar for most deviants, except for non-native non-words (D1) and content words (D4). The DLD group showed greater positivity for non-native non-words in TW1 but the opposite in TW2, whereas the TLD group showed greater positivity for content words in TW1.

For TW2 (Figure 3b), the scalp patterns showed marked differences between children and adults for all stimuli. Children’s responses presented broad central–posterior negativity with less focalised activation in the DLD than the TLD group and some frontal positive sources for all stimuli in the TLD group but only for non-words in the DLD group. Adults showed strong positive activation, broadly distributed in frontal–central areas, and less-pronounced posterior negativity for all stimulus types. 

##### Identification of MMRs in Difference Waveforms

The first ERP analysis focused on detecting statistically significant MMRs and characterising their latency and polarity. We computed four difference waveforms (DW1-4) at electrode Fz from individual ERP sets by subtracting the standard waveforms from each deviant response and then averaging each DW type across groups. To compare responses to words versus non-words, we created a “words” DW by averaging the responses to the function and content words before subtracting the standards.

**Figure 4:**
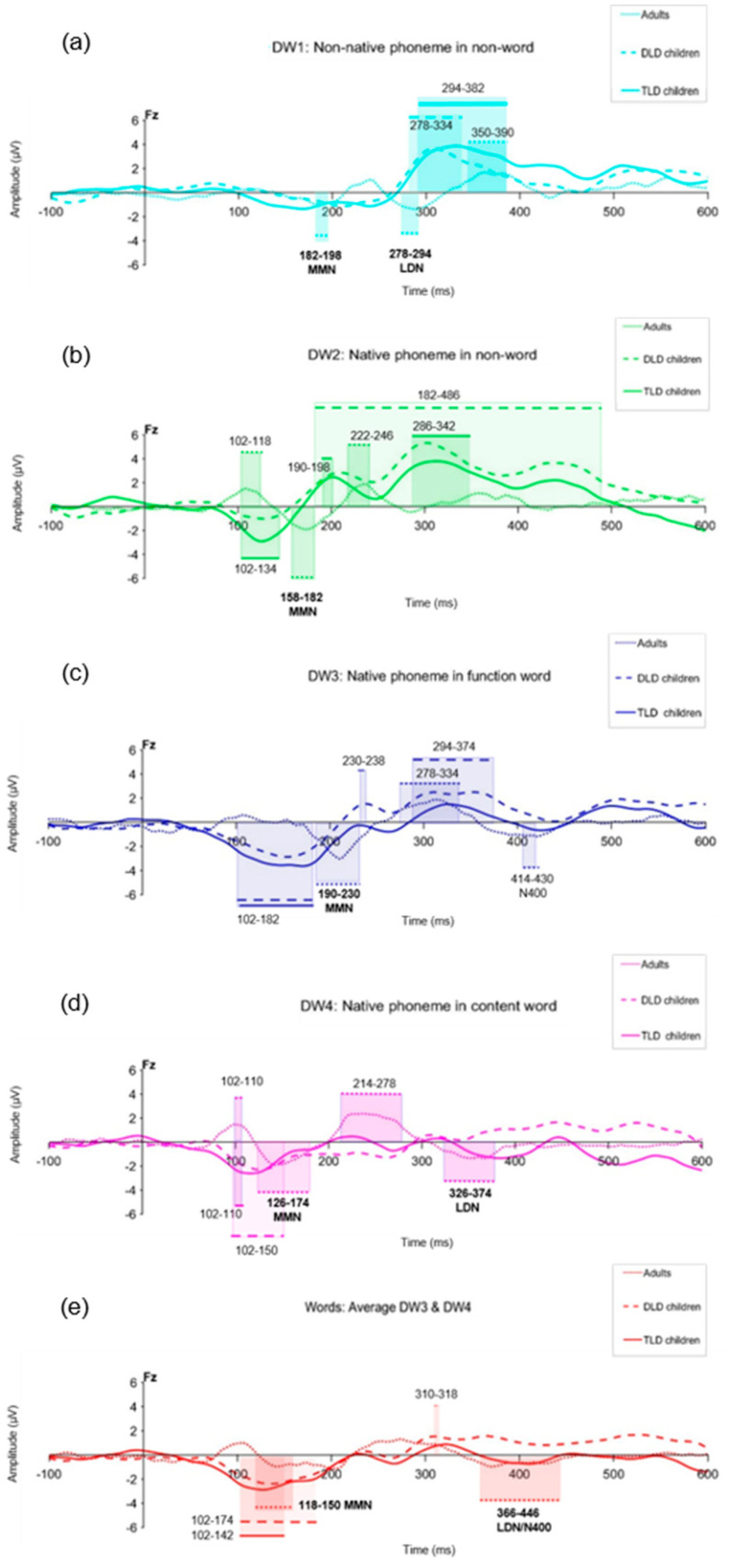
Comparison of MMRs in children and adults for each difference wave type at Fz. Continuous line: TLD children; dashed line: DLD children; dotted line: adults. Panel (**a**) DW1, non-native non-words; (**b**) DW2; native non-words; (**c**) DW3: function words; (**d**) DW4: content words; and (**e**) Words DW. Adult MMN/LDN responses are in bold type. Data low-passed filtered at 35 Hz for plotting.

The MMR statistical significance was determined using a separate Mass Univariate Analysis (MUA, [79]) for each group and stimulus type. To reduce the number of statistical comparisons, the data were downsampled to 125 Hz (bin size = 8 ms), focusing the analysis on electrode Fz in the 100–450 ms time window. MUA performed point-by-point *t*-tests and assessed their significance at the 5% level with the Benjamini and Hochberg False Discovery Rate (FDR) control procedure [82], testing the null hypothesis that a given DW has an amplitude of 0 µV against the alternative hypothesis that the DW significantly differs from 0 µV (i.e., using two-tailed tests; see Appendix A). Table 3 presents the results of the MUA, indicating the clusters of significant responses detected in all groups during the 100–450 interval, their timing, and polarity patterns in each group. Figure 4 compares the significant responses detected for each DW in all groups.

Table 3 shows significant MMRs for all DW types in all of our groups. In adults, consistent MMN responses were present for all stimulus types within 100–250 ms, but LDN responses (250–400 ms) were elicited only for non-native non-words, content words, and the combined “words”, although with a brief N400-like response to function words. In children, negative MMRs appeared early (within 200 ms), and positive ones appeared after 180 ms, but not all of the stimulus types elicited both negative and positive MMRs, as we observed in adults (except for the combined “words”). 

Negative, MMN-like responses were detected in both the TLD and DLD groups only for DWs with lexical status (function words, content words, and combined words) and in the TLD group for native non-words as well (DW2). No early negative responses were observed in either group of children for the non-native non-word contrast. Positive MMRs were present for non-words (native and non-native) in both groups, but not for stimuli with lexical status in the TLD group. In the DLD group, positive MMRs were identified for function words (DW3) and, briefly, for the combined words. Importantly, no LDN-like responses were detected in either group of children in the 250–450 ms interval.

Visual inspection of the MMRs in Figure 4 suggests similar responses for both groups of children but different patterns for adults. In general, waveform deflections in adults showed the opposite polarity, a shorter duration, and a later onset than in children, although with variable amplitude depending on the ERP peaks. In children, the amplitude and polarity were similar for all stimulus types. The onset latency for negative MMRs to words and non-words was similar between the TLD/DLD groups, but the DLD showed earlier positive responses to non-words. In terms of duration, responses in the DLD group were longer than in the DLD group.

##### Statistical Comparison between Children and Adult MMRs

The second ERP analysis focused on examining between-group differences in the MMR latency and amplitude, comparing the responses between TLD and DLD children and between children and adults in TW1 (100–250) and TW2 (250–400 ms). Peak latency was defined as the time (in ms) at which the largest negative deflection occurred in each TW, whereas the mean amplitude was calculated as the average voltage (in µV) over a 50 ms interval centred in the peak latency. Descriptive statistics for the peak latency and mean amplitude for all groups are presented in Table 4 and Table 5, respectively. 

In general, peak latency was longer in adults than in children, both for TW1 and for TW2 (Table 4). In adults, though, peak latency coincided with the significant MMN/LDN clusters, which was not observed in children. For children, peak latency values were extracted from negative deflections in TW1 and from positive deflections in TW2, regardless of whether they were significant responses or not. Thus, we performed no further analysis on peak latency measures, as the responses for all groups were not comparable.

For the mean amplitude (Table 5), children exhibited larger negative values than adults, and TLD children exhibited larger responses than DLD children in TW1. In TW2, adults showed larger negativities than children for all stimuli except for content words, and TLD children showed more negative values than the DLD group for all stimulus types.

Next, we examined the mean amplitude between-group differences for each TW using planned comparisons for each stimulus type. When Shapiro–Wilk tests confirmed a normal distribution (see Appendix B and Appendix C), we performed one-way ANOVAs, adjusting the significance level for multiple comparisons to 0.01 (0.05/5 comparisons, one per stimulus type). To account for unequal sample sizes and unequal variances (Appendix B and Appendix C), we used Tamhane’s post hoc tests, also with corrected alpha = 0.01. For non-normally distributed data, the Kruskal–Wallis test was used. Table 6 presents the results for mean amplitude between-group comparisons. In TW1, there was a significant difference in mean amplitude only for the combined-words difference wave (F(2,44) = 7.855, *p* = 0.001), and in TW2, the mean amplitude significantly differed only for native non-words (χ^2^ (2) = 15.04, *p* < 0.001), with a large effect size in both cases (η^2^ = 0. 263 and ε^2^ = 0.327, respectively). Post hoc tests for TW1 indicated significantly larger negativities for word stimuli in both groups of children (TLD M = −3.06, SD = 2.26; DLD M = −2.63 SD = 1.78) than in adults (M = −0.95, SD = 0.92). In TW2, post hoc tests showed less-negative values for native non-words in DLD children (M = 1.78, SD = 3.08) than in adults (M = −0.81, SD = 1.01), but no difference between the DLD or the adult group and TLD children (M = 0.85, SD = 3.56).

Finally, for a fine-grained comparison between TLD and DLD children, we performed MUA with the FDR control procedure [79] on each DLD-TLD DW pair. Point-by-point *t*-tests (two-tailed, q level of critical *t*-scores = 0.05) indicated no between-group amplitude differences at electrode Fz during the 100–250 ms or 250–400 ms interval (Appendix D).

#### 3.2.2. Time–Frequency Analysis of MMRs

##### Event-Related Spectral Perturbation (ERSP)

Consistent with the previous literature (e.g., [77]), all TF analyses were conducted on the parent waveforms instead of the difference waveforms. As a data quality check, we computed the spectrum of each stimulus type for each group (normalised power, µV^2^) to confirm that the 1/f pattern and typical peaks in the alpha band were present, as is expected in EEG measures reflecting cortical dynamics. All groups exhibited power increases around 10 Hz, consistent with alpha-band activity. However, only the adult and TLD groups showed additional peaks around 5 Hz and 20 Hz, which was wider in adults (to 30 Hz). 

**Figure 5:**
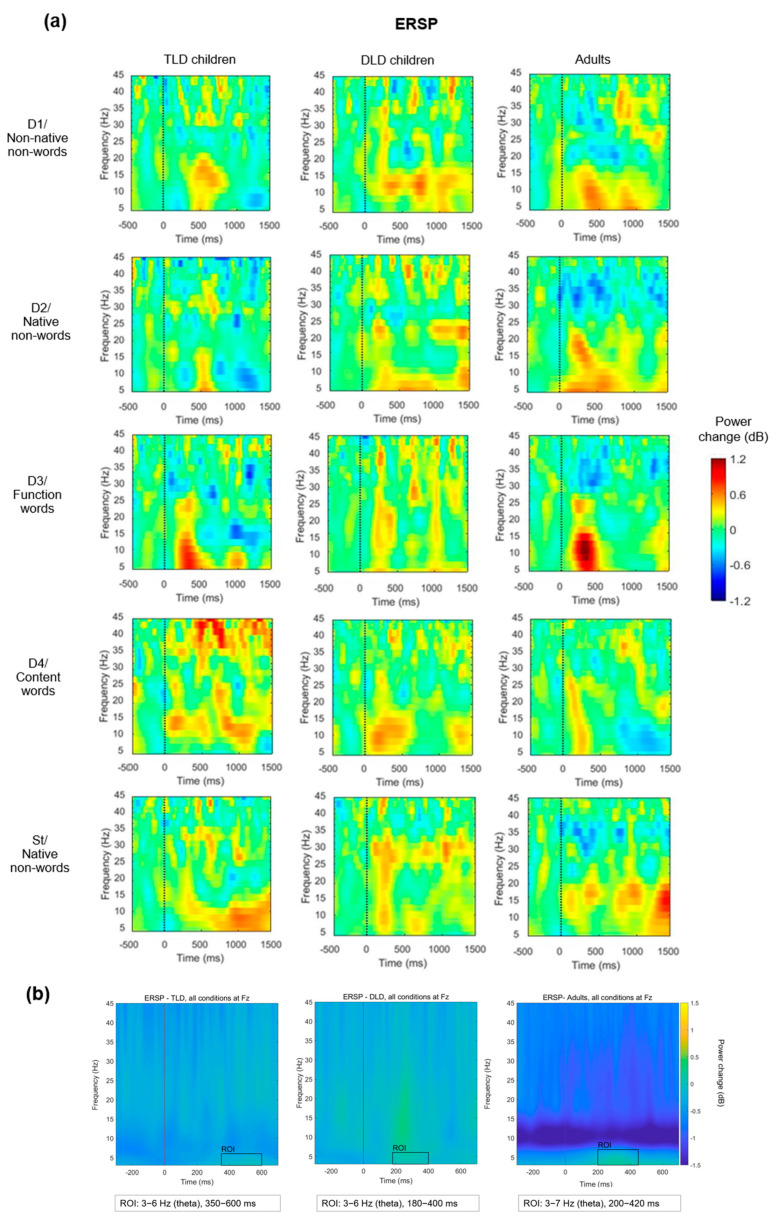
ERSP power changes (in dB) over time for all stimulus types. Panel (**a**) ERSP for all stimulus types and groups. Panel (**b**) ROIs detected in the all-condition ERSP average for TLD children (3 to 6 Hz), DLD children (3 to 6 Hz), and adults (3–7 Hz). Yellow and blue indicate power changes toward positive and negative values, respectively. Baseline correction from −500 to −200 ms.

Next, TF analysis focused on determining whether there were any between-group differences in spectral power over time for each stimulus type, as indexed by the ERSP changes. Figure 5a presents the ERSP for each stimulus type for TLD and DLD children and the adult group. To avoid a biased selection of the time windows and frequency ranges of interest for statistical analysis, for each group, we averaged the responses to all stimulus types and determined the regions of interest (ROIs) by visual inspection of the plots in a way that was blind to stimulus types (Figure 5b). 

For each group, we identified one ROI with increased activation (colour change towards yellow) in the theta band for each group: for the TLD group, between 350 and 600 ms (3–6 Hz); for the DLD group, between 180 and 400 ms (3–6 Hz); and for the adult group, between 200 and 420 ms (3–7 Hz). The onset was earlier in adults (~200 ms) and DLD children (~180 ms) than in the TLD group (~350 ms). Despite the baseline correction, in the adult group, there was a power decrease in the alpha range that spread from the start of the baseline to the post-stimulus period, suggesting an artefact affecting this frequency range. For this reason, the ERSP analysis only focused on the theta ROI.

To compare ERSP between groups, dB power was averaged across ROI time points and collapsed across frequencies to obtain the theta-band ERSP power. Table 7 presents the descriptive statistics for theta ERSP. In general, adults showed negative theta ERSP values for all stimulus types, except for function words, whereas children showed power changes towards both positive and negative values. In the TLD group, theta ERSP was similar to that in adults in magnitude and polarity for all non-words (non-native and native, including the standards), but not for word stimuli. The DLD group showed the opposite pattern, with similar responses to adults for function and content words but not for non-words. 

**Figure 6:**
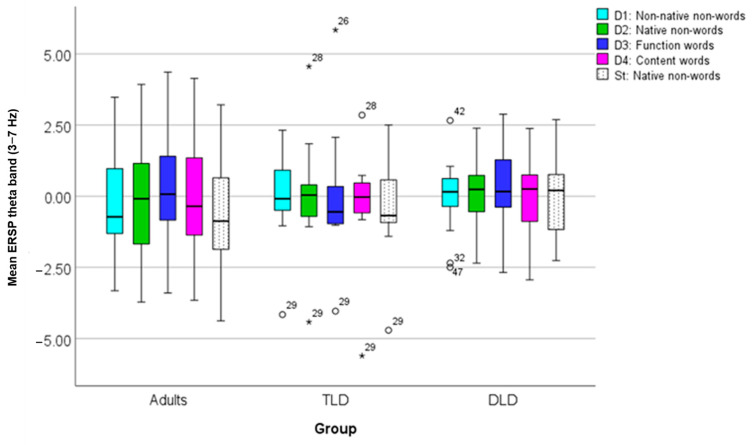
Box plots for theta ERSP (dB power) per stimulus type, all groups. ERSP baseline = −500 to −200 ms. Between-group differences are non-significant for all stimulus types at the 0.05 level.

The theta ERSP values for each group and each stimulus type are compared in Figure 6, indicating greater variability in adults than in children but a greater number of extreme values in the children’s groups, especially TLD. To quantify the differences in ERSP theta power for each stimulus type between TLD children, DLD children, and adults, we conducted a mixed repeated-measures ANOVA with “Group” as the between-subject factor and “Stimulus Type” as the within-subject factor. After checking data normality, equality of variances, and equality of covariance matrices (Appendix E), we used Greenhouse–Geisser correction to account for unmet sphericity (Mauchly’s W = 0.355, *p* < 0.001, df = 9). ERSP was normally distributed for all stimulus types, except for D4-TLD (Appendix E).

The ANOVA revealed a significant effect of stimulus type (F(2.57,113) = 3.358, *p* = 0.027), with a medium effect size (η_p_^2^ = 0.071) and adequate power = 0.70. Post hoc pairwise tests on stimulus type effects indicated a significantly larger ERSP power change for standards (M = −0.313, SD = 1.71) than for non-native non-words (M = −0.078, SD = 1.60), native non-words (M = −0.008, SD = 1.86), and function words (M = 0.185, SD = 1.95), but not for content words (M = −0.136, SD = 1.82). However, there was a non-significant effect of group (F(1,44) = 0.048, *p* = 0.953) and a non-significant group^x^stimulus type interaction (F(5.14,113) = 0.505, *p* = 0.774), both with a small effect size (η_p_^2^ = 0.002 and η_p_^2^ = 0.022, respectively) and low statistical power (0.6% and 19%, respectively).

##### Inter-Trial Phase Coherence (ITPC)

**Figure 7:**
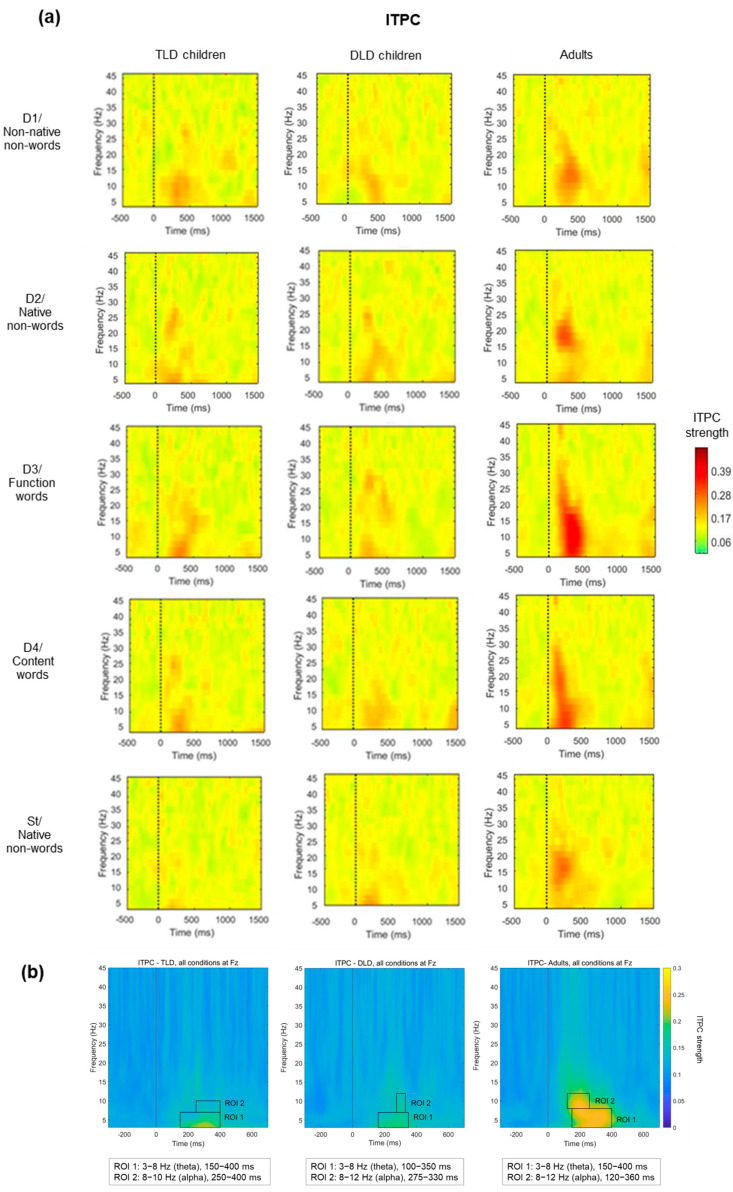
Changes in ITPC strength over time for all stimulus types. Panel (**a**) ITPC for all stimulus types and groups. Panel (**b**) ROIs detected in the all-condition ITPC average for TLD, DLD children, and adults. ROI 1: 3 to 7–8 Hz (theta band); ROI 2: 8–12 Hz (alpha band). Colour towards orange/red indicates ITPC increases over time, and green indicates phase synchrony decrease.

The final TF analysis focused on determining whether there were any between-group differences in phase coherence over time for each stimulus type, as indexed by ITPC. Figure 7a presents ITPC values for each stimulus type for TLD, DLD, and adult groups. In general, increases in ITPC appear in frequencies below 30 Hz and before (or around) 500 ms. For all stimulus types, ITPC increases are larger in adults than in both groups of children. As in ERSP measures, ROIs for ITPC statistical analysis were determined by visual inspection of the plots containing the averages of all stimulus types for each group (Figure 7b). In all the groups, it was possible to identify two ROIs in different frequency bands: ROI 1 in theta (3 to 7–8 Hz) and ROI 2 in the alpha band (8 to 10–12 Hz). 

The theta-band ITPC increase (ROI 1) had a similar onset and duration in TLD children and adults (150–400 ms), but it was slightly shorter in the DLD group (160–350 ms). The alpha-band ITPC increase (ROI 2) exhibited an earlier onset and a longer duration in adults (120–360) ms than in TLD (250–400) and DLD children (275–330), with the DLD group showing the shortest alpha ITPC increase. Table 8 presents the descriptive statistics for the theta band (ROI 1). Adults showed higher ITPC for meaningful stimuli (words than non-words) but, in general, with no difference between deviants and standard stimuli. In the TLD group, all deviants show higher ITPC than the standards. No such standard-deviant distinction is present in the DLD group.

To compare theta ITPC (ROI 1), we conducted a repeated-measures ANOVA, with “Stimulus Type” as the within-subject factor and “Group” as the between-subject factor, after checking all of the test assumptions (Appendix F). The results indicate a significant main effect of stimulus type (F(4,176) = 6.75, *p* < 0.001), with a large effect size (η_p_^2^ = 0.133) and adequate power (99%). Post hoc comparisons showed significantly higher theta ITPC for function (M = 0.22, SD = 0.094) and content words (M = 0.19, SD = 0.069) than for native non-words (M = 0.18, SD = 0.049). There was also a significant main effect of group (F(2,44) = 18.85, *p* < 0.001), with a large effect size (η_p_^2^ = 0.461) and adequate power (100%), with larger theta ITPC for adults (M = 0.23, SD = 0.061) than for the TLD (M = 0.17, SD = 0.093) and DLD (M = 0.17, SD = 0.051) groups. Finally, there was a significant stimulus type*group interaction (F(8,176) = 5.06, *p* < 0.001), with a large effect size (η_p_^2^ = 0.187) and adequate statistical power (99%). The interaction was followed up with one-way ANOVAs (Bonferroni-corrected *p* = 0.01) comparing theta ITPC between groups for each stimulus type triad. For meaningless stimuli (non-words), theta ITPC showed no significant between-group differences for non-native non-words (F(2,44) = 4.003, *p* = 0.025) or native non-words (F(2,44) = 1.194, *p* = 0.313), as well as for standard stimuli (F(2,44) = 3.117, *p* = 0.054). 

On the contrary, theta ITPC for meaningful stimuli showed significant between-group differences. For function words, theta ITPC varied significantly between groups (F(2,44) = 23.129, *p* < 0.001), with Tukey HSD post hoc comparisons indicating higher phase coherence values in adults (M = 0.310, SD = 0.082) than in the TLD (M = 0.186, SD = 0.062) and DLD (M = 0.167, SD = 0.047) groups, but with no differences between the two groups of children. Similarly, for content words (F(2,44) = 6.901, *p* = 0.002), Tukey’s HSD post hoc test showed significantly higher values in adults (M = 0.241, SD = 0.076) than in TLD (M = 0.181, SD = 0.040) and DLD (M = 0.170, SD = 0.051) children, but no differences between children’s groups. 

**Figure 8:**
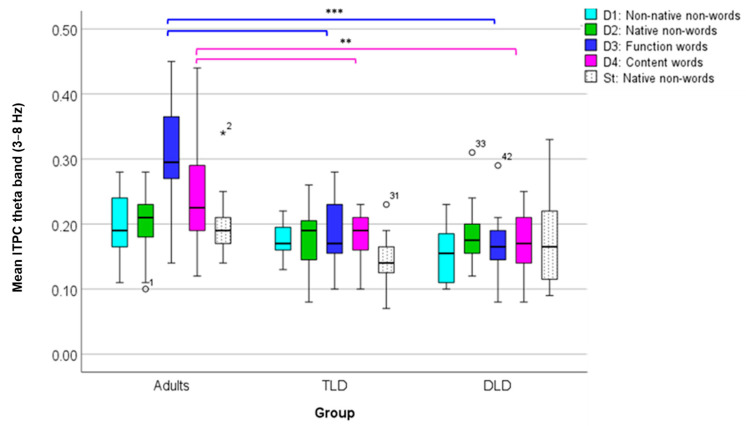
Box plots for theta-band ITPC (ROI 1) for each stimulus type, all groups. ITPC range: 0–1. (***) Significant at the 0.001 level. (**) Significant at the 0.01 level.

Figure 8 illustrates theta average ITPC values in all groups for each stimulus type. In adults, there is a marked increase in theta phase synchrony for those stimuli with lexical status (function and content words), which is not present in the groups of children. For all other stimuli, theta ITPC is rather similar within and between groups.

Next, we compared ITPC in the alpha band (ROI 2). Table 9 presents the descriptive statistics for alpha ITPC, indicating higher values for adults than for children, especially for non-words (all types). Between children’s groups, alpha ITPC values look similar for standards but are higher in the DLD than in the TLD group for non-word deviants and content words and higher in the TLD group for function words.

**Figure 9:**
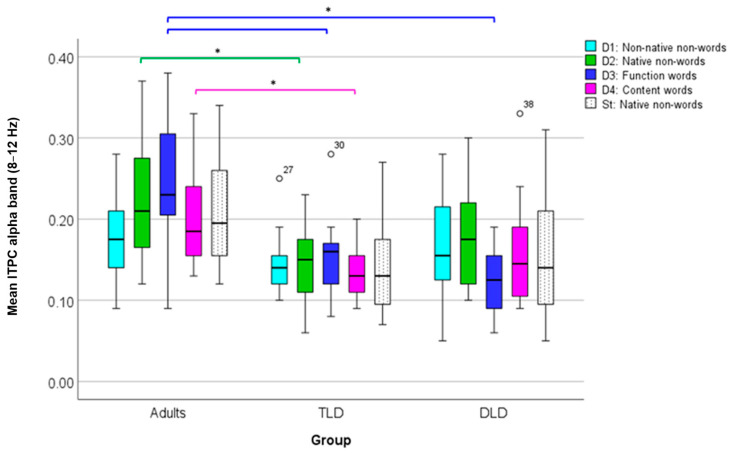
Box plots for alpha-band ITPC (ROI 2) for stimulus type, all groups. (*) Indicates significant differences at 0.01 level.

To compare changes in alpha-band ITPC (ROI 2) between groups, we conducted a repeated-measures ANOVA with the same factors as for theta after confirming that the test assumptions were met (Appendix E). The results indicated that the main effect of stimulus type was non-significant (F(4,176) = 1.073, *p* = 0.371), with a small effect size (η_p_^2^ = 0.024) and low statistical power (34%). However, there was a significant effect of group (F(2,44) = 14.84, *p* < 0.001), with a large effect size (η_p_^2^ = 0.401) and adequate power (99%), with Tamhane’s post hoc comparisons indicating significantly higher alpha ITPC in adults (M = 0.209, SD = 0.008) than in the TLD (M = 0.144, SD = 0.011) and the DLD (M = 0.156, SD = 0.009) groups at the *p* < 0.001 level. Also, a significant group^x^stimulus type interaction was detected (F(8,176) = 2.606, *p* = 0.01), with a large effect size (η_p_^2^ = 0.106) and adequate power (92%). Figure 9 compares the mean alpha ITPC for all groups. The interaction was followed up with one-way ANOVAs (Tamhane post hoc, corrected alpha = 0.01) to compare each stimulus type triad between groups. For function words, the results indicate significant between-group differences in alpha ITPC (F(2,44) = 16.902, *p* < 0.001) with a large effect size (η_p_^2^ = 0.434). Post hoc pairwise comparisons indicated higher alpha ITPC in adults (M = 0.246, SD = 0.082) than in TLD children (M = 0.156, SD = 0.056) and in DLD (M = 0.127, SD = 0.039). 

Similarly, adults showed significantly higher alpha ITPC for native non-words (M = 0.223, SD = 0.075, (F(2,44) = 5.363, *p* = 0.008)) and for content words (M = 0.197, SD = 0.051, (F(2,44) = 5.431, *p* = 0.008)) than the TLD group (M = 0.145, SD = 0.054 for native non-words; M = 0.133, SD = 0.034 for content words), with a large effect size in both cases (η^2^ = 0.196 and η^2^ = 0.198, respectively). Finally, there were no differences between adults and the DLD group. 

Taken together, the results of the TF analysis indicate an effect of group and stimulus linguistic content on MMR responses. Stimuli involving meaning (function and content words) elicited greater cortical synchrony with higher ITPC in the theta band and, to a lesser extent, in the alpha band, but the interaction indicates that this effect was only present in adults. Such effects were not detected for power change measures (ERSP).

#### 3.2.3. Correlation between Phonological Awareness and EEG Measures

Finally, we examined whether there was an association between children’s phonological awareness skills and EEG measures in each group separately. To account for missing behavioural data and the reduced sample size (TLD, n = 8 and DLD, n = 14 after pairwise case deletion), Spearman’s rank correlation (non-parametric) was computed to assess the relationship between phonological awareness (PECFO) and EEG measures: mean amplitude in the MMN and LDN intervals (TW1 and TW2, respectively) and ERPS and ITPC (for ROI 1 and ROI 2). The results of the correlation analysis for the TLD and DLD groups are displayed in Table 10. After Bonferroni correction (alpha = 0.002) was applied, there were no significant correlations in either group between phonological awareness scores and any EEG measure.

## 4. Discussion

The present study investigated the cortical discrimination of speech contrasts with varying linguistic complexity in young children with and without DLD and in adults. We were interested in whether top-down processing modulates cortical speech perception in children, as we had previously confirmed in adults. Cortical speech discrimination was measured via ERP and a time–frequency analysis of the MMR. As hypothesised, we detected significant MMRs to all speech stimuli in both groups of children and in adults, with different patterns between children and adults in terms of polarity, mean amplitude, and ITPC. However, contrary to our expectations, we found no differences between TLD and DLD children on any of the EEG measures, nor a correlation with behavioural tests of phonological abilities, although the phonological awareness scores were significantly lower in the DLD than in the TLD group. Finally, only adults were sensitive to manipulations in the stimulus linguistic content, with more robust MMNs for higher-order representations (words versus non-words). These results suggest that top-down language modulations in speech perception are present in adulthood, but they are not developed yet in early childhood, as children did not show enhanced responses to contrasts with lexical status. Alternatively, such top-down modulations may be present but not detectable with the current ERP paradigm in these groups of children. Importantly, we found no evidence of impaired speech processing in DLD at the cortical level, indicating that speech perception in children with DLD and TLD could be similar, although such null results should be interpreted cautiously.

Importantly, our results confirm that all of our stimulus types elicited at least one significant negative or positive MMR for all our groups. However, a key difference between the children and the adult group was the lack of significant early MMNs in children for most non-words and later LDNs for all stimulus types. These findings contradict the previous literature reporting the early presence of the MMN [30] and greater LDNs in children than in adults [13]. For example, Kuuluvainen et al. [39] reported that different speech and nonspeech contrasts elicited significant MMNs between 200 and 350 ms and LDNs for the 350-500 interval. However, the children in their study were 6–7 years old, thus older than our participants, and their speech stimuli did not include a change in meaning. An alternative explanation for the lack of MMNs/LDNs relates to a possible attenuation effect of an extended ISI duration. In young children, the MMR reflects sensory memory capacity, which has been reported to increase between the ages of 2 and 6 years, resulting in progressively better discrimination of memory traces with longer delays, that is, ISIs over 500 ms [84]. In our experiment, we used an ISI of 1000 ms to avoid neural refractoriness [16]; however, this could have had a detrimental effect on eliciting the MMN/LDN.

As predicted, both groups of children exhibited immature responses when compared to adults, as indicated by positive MMRs during the MMN and LDN intervals. The previous literature has described a positive polarity for MMRs in infants [31] and for children until the age of 7 years in response to complex stimuli [24]. On the contrary, other studies in preschoolers [39,43] indicate that one could expect typical, adult-resembling MMN/LDN patterns, even if occurring at longer latencies. However, we observed positive polarity only for non-words, indicating that meaningless word forms elicited more immature responses than words, maybe because the lack of meaning makes them more complex to perceive. This interpretation contradicts the possible lack of top-down language effects discussed earlier but could be explained by the fact that point-by-point analysis reveals differences that are no longer detectable when averaging values across a time window (e.g., because they are cancelled out). In addition, scalp patterns in children showed broad distributions, especially for the later time window (TW2), instead of the more focalised responses often seen in adults [30], also indicating less mature MMRs. 

Another indicator of immaturity is that children exhibited greater MMR latency and a longer duration (for example, a 164 ms long MMR to native non-words in the DLD group), making it hard to differentiate between early and late MMRs to some stimuli and to compare latencies between groups, particularly for non-words. In the literature, responses in 3-year-olds for monosyllabic function words differing in their final consonant peaked at 262 ms after deviance [28]. Strotseva-Feinschmidt et al. [44] reported latencies between 180 and 350 ms in children between 5 and 8 years, whereas Paquette et al. [27] showed MMRs to phonemic contrasts peaking at 272 ms in 3- to 7-year-old children. These findings are consistent with the MMR latency and duration we observed for non-words but occur much later than the responses we detected for function and content words. Again, this could indicate easier and faster cortical processing of meaningful stimuli. For phoneme or word deviants, Strotseva-Feinschmidt et al. [44], who used similar stimuli to those in our study (contrasts between monosyllabic function words) in the same age group, reported overall latencies of 400 ms for the MMN and 700 ms for the LDN, which are much longer. However, the use of peak measures for ERPs may be suboptimal, as they are sensitive to the noise level [79], which can be high in paediatric EEG [85]. 

Regarding the mean MMR amplitude, our findings are partially consistent with the previous literature. For example, Paquette et al. [27] reported an amplitude of −0.067 µV at Fz for phonemic contrasts in 3–7-year-old children, which agrees with our results for non-words in TW2 but not in TW1, in which children showed much larger negative values. The lack of amplitude differences between the two groups of children is consistent with multiple studies failing to differentiate between TLD and DLD groups based on speech-elicited MMRs [20]. When comparing children versus adults, our findings support our prediction of significantly smaller amplitudes in adults than in children, but this occurred only for words in TW1. Contrary to our hypothesis, the mean amplitude was smaller in children than in adults, but only for the DLD group in TW2 for native non-words. This resembles previous findings of smaller MMN amplitudes for 6-year-old children [69].

In terms of TF analysis, we confirmed less synchronised activity in children than in adults, but only for words and when measured by ITPC but not by ERSP. Theta ERSP was affected by the linguistic content (stimulus type), with reduced power change for standards than for most deviants (except function words), which is consistent with the idea of increased theta synchronisation for novel stimuli. Larger ERSP for deviants is consistent with the findings reported by Fuentemilla et al. [58] and Hsiao et al. [59,60]. The lack of power differences we observed between standards and function words could be explained by a larger ERP negativity; thus, the lack of a significant effect may result from acoustic differences. However, if this is the case, we would expect to see a consistent pattern for function words throughout all measures that was not present, for example, increased amplitude with higher ERSP and ITPC. Importantly, there were no ERSP differences between groups of children or between children and adults, contradicting our predictions but in line with the findings reported by Bishop and colleagues [13]. Also, it is worth considering some methodological issues in our ERSP analysis: (i) determining ROIs by visual inspection of the condition average plots was suboptimal, as despite baseline correction, the adult plot presented unexplained pre-stimulus negative alpha power, suggesting a remaining artefact; (ii) for most analyses, the effect size was small, except for ITPC, meaning that some between-group differences may have gone undetected; and (iii) group mean values for ERSP and ITPC could have been affected by the presence of extreme values observed in the groups of children.

On the contrary, ITPC in the theta band (and, to a lesser extent, in alpha) showed a main effect of linguistic content and language status, with a significant interaction between them, all with a large effect size. This is a key finding, as it indicates higher synchrony in adults than in children, but only for meaningful stimuli (function and content words), consistent with our hypothesis of greater top-down language modulations for higher-order linguistic representations in participants with more advanced language skills. Previous research has linked increased theta ITPC to syllable encoding and discrimination [86], whereas alpha ITPC is thought to reflect the automatic allocation of attentional resources for speech sounds and the inhibition of task-irrelevant stimuli [87]. The presence of robust ITPC differences indicates more efficient responses to speech in adults than in children, which is in-line with previous studies. This aligns with Skeide and Friederici’s [19] proposal of greater bottom-up modulations and the slow emergence of top-down modulations after the age of six years. However, as there is no difference between TLD and DLD children, it could be argued that our results come from brain maturational changes (effect of age) rather than top-down influences based on linguistic abilities. 

Finally, phonological awareness test scores did not correlate with any EEG measure, even though significantly lower scores were observed in the DLD than in the TLD group. This contradicts previous evidence of better phonological skills associated with larger MMNs for phonemic changes in 5-to 6-year-old children [50], but it is consistent with many studies that report no clear links between ERPs and behavioural measures.

To our knowledge, this is the first study to use a multifeature paradigm in Spanish-speaking preschoolers with a DLD diagnosis and to compare their responses to age-matched TLD controls and adults. Moreover, few MMR studies in children have used not only syllables or non-words but also words and validated an experiment in a previous study to obtain reference adult response patterns. One contribution of this study is that we confirmed that our multifeature experiment was able to elicit robust MMRs in young children presenting multiple speech deviants while reducing the EEG testing time. In less than 20 min (plus set-up times), it was possible to collect enough clean data for all of the children who underwent the EEG session, as demonstrated by noise levels under 35% for all participants and stimulus types. Combining artefact rejection and correction procedures, we were able to include the data of all participants, with a minimum of 42 trials per stimulus type, which is well above the standard threshold for paediatric studies (10 trials, according to [22]). This highlights the importance of combining manual and automatic data-cleaning procedures to improve data quality [56], helping to reduce data loss and sampling bias due to participant exclusion [22].

Retaining all of the participants was especially important for this study, as one of the main limitations was the small sample size of each group, which was a consequence of the COVID-19 pandemic restrictions for data collection. Although small samples are not uncommon in child EEG studies because of difficult recruitment and high drop-out rates [18], especially for clinical groups, it is worth noting that the reduced number of child participants may have affected the statistical power of our results. This is a relevant aspect to consider in paediatric EEG studies, as even children with typical development show high inter-individual variability, which makes it harder to detect differences between TLD and DLD children. Importantly, differences in language and nonverbal cognitive skills within the DLD group could have influenced the MMR results. Although all language-impaired children in this study had an expressive-receptive disorder diagnosis, it was impossible to determine the exact level of homogeneity in their language development and cognitive profiles. Although we did not perform a full nonverbal assessment, it is noteworthy that the DLD group performed poorer than the TLD group in the Block Design screening task (although within the normal range), but these scores were not included as a covariate in our statistical analysis. If these differences arise from poorer general cognitive skills in DLD children, this could have introduced higher, uncontrolled variability in the MMR, impeding a distinction between the DLD and TLD groups based on their cortical activity. Importantly, a large body of evidence indicates that the language and cognitive symptoms in DLD are dynamic over time [88,89], suggesting that identifying neural markers of language outcomes at the group level could be more challenging than expected.

A second limitation is that our stimuli differed in their acoustic structure. Acoustical matching of the initial phonemes for non-words was considerably easier than for word stimuli, as they should also be matched in their age of acquisition and oral frequency. Thus, larger acoustic differences rather than effects of language knowledge or linguistic content may have driven some of our results, as in Lee et al. [90], who reported negative, adult-like MMRs to larger syllabic deviants and positive MMRs to small deviants. However, if this were the case, we would expect consistency between the different EEG measures; for example, greater MMR amplitude should coincide with greater ERSP and ITPC for the same type of stimulus, which we did not find.

An important remaining question is which EEG analyses are more suitable when comparing cortical speech perception responses between groups of children and children versus adults, given the high diversity of latencies and amplitude values, electrodes, and time windows reported in the previous literature. This complicates the a priori selection of time windows and electrodes for analysis, as findings vary substantially across studies. A possible approach to reduce bias in our ERP analysis is to follow the same steps used in the TF analysis for determining ROIs for amplitude and latency, for example, using global field power from the total group average, as in François et al. [91]. Nevertheless, a contribution of this study is that it confirms that ITPC is a robust measure, probably a more suitable one for comparing children and adults, chiefly because the ITPC results showed large effect sizes, which was not the case for the ERSP and ERP measures. Moreover, theta ITPC increases were independent of changes in amplitude or power, as the MMN amplitude was not larger for adults than for children, and the ERSP showed no between-group differences. Thus, our findings corroborate the value of TF analysis as a relevant complement to ERP measures, encouraging its use in further speech perception development studies. However, it is important to note that ITPC distortions may also occur due to the effects of noise or a small ERP amplitude [92]. 

Another remaining challenge is the selection of the most appropriate statistical methods for group-level comparisons. Considering the within-group variability in the EEG and some missing data in the phonological awareness measures in our study, using linear mixed-effects (LME) models with participants as random effects was a plausible alternative. However, our data did not meet the assumptions for LME models, nor did the LME model perform better than the ANOVA in terms of model fit. Thus, the repeated-measures ANOVA was the most appropriate model for our data, even though it does not address variability as well as the LME does. In addition, some of our variables were not normally distributed (especially in the ITPC analysis), but due to a lack of more appropriate tests to compare groups, we still used the repeated-measures ANOVA. Although this is a robust test, it is noteworthy this could be a possible reason for the observed differences between adults and children in ITPC. Importantly, these difficulties in meeting the test assumptions indicate that linear methods may not always be optimal for developmental EEG data, especially when including clinical groups. 

Future research in TLD/DLD groups could explore other EEG measures related to speech perception, such as resting-state analysis or the linear modelling of continuous speech tracking, helping to increase the ecological validity of the experiments. In addition, behavioural testing could be extended to include morphological and lexical tests to determine whether there is an association between these language skills and EEG measures. Another possibility is to replicate this study in groups of older or younger children or, ideally, in a longitudinal follow-up study to re-test these same children at a later age. This was initially considered for this study; however, the data collection process for this study was severely disrupted by the COVID-19 pandemic, impeding us from conducting a second testing phase in children from this school that was planned for 2020-21. In older children, we might be able to detect more pronounced differences in the MMR between TLD and DLD children as their cortical activity becomes more consistent with age and, potentially, the emergence of top-down language effects, at least in the TLD group. Likewise, other statistical methods could be more informative than MUA or ANOVA to address the multivariate nature of the EEG [93]. For example, a multivariate pattern analysis (MVPA) could determine whether children can be correctly classified based on individual EEG measures [94].

## 5. Conclusions

Taken together, these findings confirm that the adult group showed more consistent speech-processing responses than children, but in children, this was not determined by their typical or atypical language status. Importantly, the fact that adults showed greater ITPC in theta (and alpha) bands for function and content words indicates that they may detect phonemic changes better than children, but they do so when these contrasts are contained in meaningful word forms and not in non-words. The lack of top-down language effects on the TLD/DLD groups suggests that these emerge at some point in childhood, although later than the age range we studied (although it could also be explained by the characteristics of our sample and stimuli). Thus, future studies could explore language modulations in speech processing in TLD/DLD children at older ages, for example, late childhood or adolescence.

## Figures and Tables

**Table 1:** Participants’ age, hearing levels, and nonverbal intelligence scores.

Measure	TLD (n = 11)			DLD (n = 16)			Adults (n = 20)		
	M	SD	Min–Max	M	SD	Min–Max	M	SD	Min–Max
Age (years.months)	5.2	0.23	4.10–5.6	5.2	0.33	4.9–5.7	34.2	4.8	24.9–44.11
PTA left ear (dB HL)	20.9	1.69	20–25	20.6	0.91	20–22.5	6.9	3.6	0–13
PTA right ear (dB HL)	21.3	1.58	20–25	20.6	1.12	20–23.8	6.2	3.7	0–13
Block Design (Scaled score)	18.1	1.58	15–19	15.7	2.98	10–19	60.7	7.8	44–79

Note. Group mean values (M), standard deviation (SD), and minimum/maximum values for age, pure tone audiometry (PTA), and the Block Design test.

**Table 3:** Significant ERP responses to all DW types detected between 100 and 450 ms.

Group	TLD			DLD			Adults		
DW Type	Significant Responses (ms)	Duration (ms)	Polarity	Significant Responses (ms)	Duration (ms)	Polarity	Significant Responses (ms)	Duration (ms)	Polarity
DW1/ Non- native non-words	-- -- 294–382	-- -- 88	-- -- Pos.	-- -- 278–334	-- -- 56	-- -- Pos.	**182–198** 278–294 350–390	24 16 40	Neg. Neg. Pos.
DW2/ Native non-words	102–134 -- 190–198 286–342	32 -- 8 56	Neg. -- Pos. Pos	-- -- 182–486 --	-- -- 164 --	-- -- Pos. --	102–118 **158–182** 222–246 --	16 24 24 --	Pos. Neg. Pos. --
DW3/ Function words	102–182 -- -- --	80 -- -- --	Neg. -- -- --	102–182 230–238 294–374 --	80 8 80 --	Neg. Pos. Pos. --	-- **190–230** 278–334 414–430	-- 40 56 16	-- Neg. Pos. Neg.
DW4/ Content words	102–110 -- -- --	8 -- -- --	Neg. -- -- --	102–150 -- -- --	48 -- -- --	Neg. -- -- --	102–110 **126–174** 214–278 326–374	8 48 64 48	Pos. Neg. Pos. Neg.
Words (function + content)	102–142 -- --	40 -- --	Neg. -- --	102–174 310–318 --	72 8 --	Neg. Pos. --	**118–150** -- 366–446	32 -- 80	Neg -- Neg

Note. Adult MMN responses are marked in bold type, and LDN responses are underlined.

**Table 4:** Descriptive statistics (means, M, and standard deviation, SD) for MMR peak latency (ms) for all groups in TW1-TW2.

			**TW1**			
	**TLD**		**DLD**		**Adults**	
	**M**	**SD**	**M**	**SD**	**M**	**SD**
DW1/Non-native non-words	184.6	50.71	197.1	37.9	178.5	29.8
DW2/Native non-words	136.6	38.5	127.9	22.2	167.4	22.5
DW3/Function words	145.1	23.2	160.1	28.5	202.5	23.2
DW4/Content words	137.1	38.9	161.3	51.0	157.0	21.3
DW Words	131.8	21.7	160.0	38.0	154.4	35.7
			**TW2**			
	**TLD**		**DLD**		**Adults**	
	**M**	**SD**	**M**	**SD**	**M**	**SD**
DW1/Non-native non-words	340.9	35.6	316.9	28.6	323.3	62.4
DW2/Native non-words	310.0	21.7	314.0	28.5	335.4	64.2
DW3/Function words	337.3	32.7	329.4	32.0	364.9	65.1
DW4/Content words	321.6	42.3	338.6	42.0	371.2	41.4
DW Words	326.6	32.9	330.4	34.2	400.1	24.1

**Table 5:** Descriptive statistics (means, M, and standard deviation, SD) for mean MMR amplitude (µV), all groups in TW1–TW2.

			**TW1**			
	**TLD**		**DLD**		**Adults**	
	**M**	**SD**	**M**	**SD**	**M**	**SD**
DW1/Non-native non-words	−2.35	2.73	−1.57	2.16	−0.76	1.13
DW2/Native non-words	−2.45	2.55	−1.19	2.02	−1.59	1.51
DW3/Function words	−3.97	3.22	−3.16	2.55	−2.08	0.92
DW4/Content words	−2.72	2.62	−2.60	1.68	−1.58	1.11
DW Words	−3.06	2.26	−2.63	1.78	−0.95	0.92
			**TW2**			
	**TLD**		**DLD**		**Adults**	
	**M**	**SD**	**M**	**SD**	**M**	**SD**
DW1/Non-native non-words	−0.81	2.80	−0.29	2.77	−1.45	1.48
DW2/Native non-words	0.85	3.56	1.78	3.08	−0.81	1.01
DW3/Function words	−1.66	3.55	−0.03	2.17	−1.85	1.88
DW4/Content words	−2.27	3.07	−1.67	2.30	−1.56	1.55
DW Words	−1.58	2.50	−0.43	1.67	−1.72	1.68

**Table 6:** Results for mean amplitude comparisons between groups.

	**Test**	**Test Statistic**		***p***	**Effect Size**	
**TW1**		**F**	**χ^2^**		**η^2^**	**ε^2^**
DW1/Non-native non-words	One-way ANOVA	2.441	-	0.099	0.100	-
DW2/Native non-words	Kruskal–Wallis	-	1.71	0.426	-	0.037
DW3/Function words	One-way ANOVA	2.277	-	0.115	0.094	-
DW4/Content words	One-way ANOVA	2.139	-	0.130	0.089	-
DW Words	One-way ANOVA	7.855	-	0.001 (*)	0.263	-
**TW2**		**F**	**χ^2^**	***p***	**η^2^**	**ε^2^**
DW1/Non-native non-words	One-way ANOVA	1.141	-	0.329	0.049	-
DW2/Native non-words	Kruskal–Wallis	-	15.04	<0.001 (*)	-	0.327
DW3/Function words	One-way ANOVA	2.727	-	0.076	0.110	-
DW4/Content words	Kruskal–Wallis	-	0.47	0.790	-	0.010
DW Words	Kruskal–Wallis	-	2.56	0.278	-	0.056

Note. For all tests, ANOVA df = (2, 44); Kruskal–Wallis df = 2. (*), Significant at the 0.01 level.

**Table 7:** Descriptive statistics for theta-band average ERSP (dB power) for all groups.

	TLD		DLD		Adults	
	M	SD	M	SD	M	SD
D1/Non-native non-words	−0.109	1.65	0.001	1.25	−0.125	1.88
D2/Native non-words	0.039	2.15	0.008	1.27	−0.072	2.16
D3/Function words	0.032	2.43	0.257	1.63	0.267	2.01
D4/Content words	−0.326	2.03	−0.021	1.39	−0.063	2.07
St/Native non-word	−0.428	1.81	−0.041	1.34	−0.471	1.97

Note. Measures on parent waveforms (not subtracted).

**Table 8:** Descriptive statistics for average theta ITPC (ROI 1), all groups.

	TLD		DLD		Adults	
	Mean	SD	Mean	SD	Mean	SD
D1/Non-native non-words	0.174	0.027	0.154	0.047	0.196	0.049
D2/Native non-words	0.175	0.052	0.180	0.046	0.200	0.050
D3/Function words	0.186	0.062	0.167	0.046	0.308	0.082
D4/Content words	0.181	0.040	0.170	0.051	0.241	0.076
St/Native non-word	0.147	0.043	0.172	0.067	0.196	0.046

**Table 9:** Descriptive statistics for average alpha ITPC (ROI 2), all groups.

	TLD		DLD		Adults	
	M	SD	M	SD	M	SD
D1/Non-native non-words	0.146	0.043	0.166	0.065	0.175	0.047
D2/Native non-words	0.146	0.053	0.176	0.061	0.223	0.075
D3/Function words	0.156	0.054	0.127	0.040	0.246	0.082
D4/Content words	0.134	0.034	0.159	0.065	0.197	0.052
St/Native non-word	0.143	0.065	0.154	0.074	0.205	0.065

**Table 10:** Correlation between phonological awareness and EEG measures per group.

			**ERP Measures**				
			**DW1/** **Non-Native Non-Words**	**DW2/** **Native** **Non-Words**	**DW3/** **Function Words**	**DW4/** **Content Words**	**DW** **Words**
	Mean amplitude TW1						
PECFO	TLD	r	−0.262	−0.024	−0.143	−0.143	−0.381
		*p*	0.531	0.955	0.736	0.736	0.352
	DLD	r	0.177	0.044	−0.11	0.108	0.071
		*p*	0.545	0.881	0.707	0.713	0.81
	Mean amplitude TW2						
	TLD	r	−0.548	0.333	0.095	−0.024	−0.024
		*p*	0.16	0.42	0.823	0.955	0.955
	DLD	r	0.212	−0.411	−0.323	−0.135	−0.358
		*p*	0.467	0.144	0.261	0.646	0.209
		**Time–Frequency Measures**					
			**D1/Non-Native Non-Words**	**D2/** **Native** **Non-Words**	**D3/** **Function Words**	**D4/Content Words**	**St/** **Native** **Non-Words**
PECFO	ERSP theta						
	TLD	r	0.167	−0.286	−0.095	−0.024	−0.119
		*p*	0.693	0.493	0.823	0.955	0.779
	DLD	r	−0.02	−0.476	−0.362	-0.429	−0.557
		*p*	0.946	0.086	0.203	0.126	0.039
	ITPC theta (ROI 1)						
	TLD	r	0.503	−0.359	−0.241	0.386	−0.659
		*p*	0.204	0.382	0.565	0.346	0.076
	DLD	r	−0.047	0.011	−0.287	0.278	−0.36
		*p*	0.873	0.97	0.319	0.336	0.206
	ITPC alpha (ROI 2)						
	TLD	r	0.289	−0.071	−0.195	0.723	−0.422
		*p*	0.487	0.867	0.643	0.043	0.298
	DLD	r	0.21	−0.327	0.102	−0.318	−0.195
		*p*	0.471	0.254	0.728	0.268	0.504

Note. r = Spearman’s rho; *p* = 2-tailed significance value. Corrected alpha = 0.002. TLD df = 6; DLD df = 12.

## Data Availability

The data generated and analysed in this study are available on reasonable request from the corresponding author. The data are not publicly available as they are human data from adults and children in neurotypical and clinical groups.

## Appendix A

**Table A1:** MUA results for MMR identification at Fz, all groups.

	TLD			DLD			Adults		
	Critical *t*-Scores	Test-Wise Alpha	Upper-Bound FDR	Critical *t*-Scores	Test-Wise Alpha	Upper-Bound FDR	Critical *t*-Scores	Test-Wise Alpha	Upper-Bound FDR
Non-Nat Non-words	−3.24/ 3.24	0.009	0.6	−3.25/ 3.25	0.005	0.4	−2.85/2.85	0.01	0.6
Native non-words	−3.07/ 3.07	0.012	0.7	−2.78/ 2.78	0.014	2.0	−2.80/2.80	0.01	0.7
Function Words	−3.19/ 3.19	0.010	0.6	−2.81/ 2.81	0.013	1.1	−2.69/2.69	0.02	0.9
Content Words	--	0.002	--	−3.34/ 3.34	0.005	0.4	−2.41/2.41	0.03	1.4
Words	−3.74/ 3.74	0.004	0.3	−2.98/ 2.98	0.010	0.6			

Note: TLD/DLD time window: 100–550 ms; adults’ time window: 50–550 ms. TLD group, df = 10; DLD group, df = 15; adults’ df = 19.

## Appendix B

**Table A2:** ANOVA assumption check for the variable mean amplitude (TW1).

**Mean Amplitude (100–250 ms, Peak-Centred)**								
**Homogeneity of Variance ^a^**				**Normality ^b^**				
**DW Type**	**Levene st.**	**df1**	**df2**	***p***	**Group**	**Shap.–Wilk st.**	**df**	***p***
DW1	3.7	2	44	**0.033**	Adults	0.943	20	0.277
					TLD	0.975	11	0.935
					DLD	0.92	16	0.168
DW2	0.429	2	44	0.654	Adults	0.97	20	0.761
					TLD	0.79	11	**0.007**
					DLD	0.939	16	0.336
Words	4.659	2	44	**0.015**	Adults	0.927	20	0.133
					TLD	0.929	11	0.398
					DLD	0.98	16	0.966
DW3	5.104	2	44	**0.01**	Adults	0.922	20	0.109
					TLD	0.865	11	0.066
					DLD	0.946	16	0.436
DW4	5.046	2	44	**0.011**	Adults	0.981	20	0.943
					TLD	0.922	11	0.334
					DLD	0.933	16	0.276
					Phonological Awareness Test			
					TLD	0.873	8	0.162
					DLD	0.909	14	0.151
**Within-Subject Effect: Stimulus Type**								
**Equality of Covariance Matrices ^c^**					**Sphericity ^d^**			
**Box’s M**	**F**	**df1**	**df2**	***p***	**Mauchly’s W**	**Approx. Chi^2^**	**df**	***p***
93.16	2.538	30	3795	**<0.001**	0.181	72.46	9	**<0.001**

Note. Significant tests are indicated in bold fonts. ^a^ Tests the null hypothesis of equal variances across groups. ^b^ Tests the null hypothesis of a normal data distribution. ^c^ Tests the null hypothesis of equal observed covariance matrices of the dependent variables (DVs) across groups. ^d^ Tests the null hypothesis that the error covariance matrix of the orthonormalised transformed DVs is proportional to an identity matrix.

## Appendix C

**Table A3:** ANOVA assumption check for the variable mean amplitude (TW2).

**Mean Amplitude (250–400 ms, Peak-Centred)**								
**Homogeneity of Variance ^a^**				**Normality ^b^**				
**DW Type**	**Levene st.**	**df1**	**df2**	***p***	**Group**	**Shap.–Wilk st.**	**df**	***p***
DW1	4.087	2	44	0.024	Adults	0.929	20	0.151
					TLD	0.916	11	0.288
					DLD	0.983	16	0.981
DW2	4.533	2	44	0.016	Adults	0.979	20	0.920
					TLD	0.910	11	0.242
					DLD	0.878	16	**0.036**
Words	1.048	2	44	0.359	Adults	0.857	20	**0.007**
					TLD	0.767	11	**0.003**
					DLD	0.959	16	0.645
DW3	3.070	2	44	0.056	Adults	0.961	20	0.574
					TLD	0.970	11	0.884
					DLD	0.939	16	0.340
DW4	4.675	2	44	0.014	Adults	0.849	20	**0.005**
					TLD	0.937	11	0.489
					DLD	0.946	16	0.433
**Within--Subject Effect: Stimulus Type**								
**Equality of Covariance Matrices ^c^**				**Sphericity ^d^**				
**Box’s M**	**F**	**df1**	**df2**	***p***	**Mauchly’s W**	**Approx. Chi^2^**	**df**	***p***
109.75	2.99	30	3795	**<0.001**	0.240	60.53	9	**<0.001**

Note. Significant tests are indicated in bold fonts. ^a^ Tests the null hypothesis of equal variances across groups. ^b^ Tests the null hypothesis of a normal data distribution. ^c^ Tests the null hypothesis that the observed covariance matrices of the DVs are equal across groups. ^d^ Tests the null hypothesis that the error covariance matrix of the orthonormalised transformed DVs is proportional to an identity matrix.

## Appendix D

**Table A4:** MUA results for MMR amplitude comparisons in TLD/DLD children.

	100–250 ms	250–400 ms
	All FDR Adjusted *p*-Values ≥	All FDR Adjusted *p*-Values ≥
Non-native non-words	0.869	0.853
Native non-words	0.275	0.492
Function words	0.558	0.404
Content words	0.725	0.618
Words	0.978	0.478

Note: *t*-score df = 25; total comparisons = 19 (number of time points, exact boundaries 102–246 and 246–398 ms).

## Appendix E

**Table A5:** ANOVA assumption check for the variable ERSP.

**ERSP**								
**Homogeneity of Variance ^a^**				**Normality ^b^**				
**Stimulus Type**	**Levene st.**	**df1**	**df2**	***p***	**Group**	**Shap.–Wilk st.**	**df**	***p***
D1	2.043	2	44	0.142	Adults	0.940	20	0.235
					TLD	0.881	11	0.108
					DLD	0.919	16	0.163
D2	1.657	2	44	0.202	Adults	0.965	20	0.658
					TLD	0.894	11	0.157
					DLD	0.922	16	0.180
D3	0.274	2	44	0.762	Adults	0.959	20	0.519
					TLD	0.859	11	0.056
					DLD	0.955	16	0.573
D4	1.033	2	44	0.364	Adults	0.969	20	0.726
					TLD	0.788	11	**0.007**
					DLD	0.963	16	0.711
St	1.005	2	44	0.374	Adults	0.955	20	0.441
					TLD	0.903	11	0.203
					DLD	0.960	16	0.661
**Within-Subject Effect: Stimulus Type**								
**Equality of Covariance Matrices ^c^**					**Sphericity ^d^**			
**Box’s M**	**F**	**df1**	**df2**	***p***	**Mauchly’s W**	**Approx. Chi^2^**	**df**	***p***
69.32	1.89	30	3795	**0.002**	0.355	43.91	9	**<0.001**

Note. Significant tests are indicated in bold fonts. ^a^ Tests the null hypothesis of equal variances across groups. ^b^ Tests the null hypothesis of a normal data distribution. ^c^ Tests the null hypothesis that the observed covariance matrices of the dependent variables are equal across groups. ^d^ Tests the null hypothesis that the error covariance matrix of the orthonormalised transformed dependent variables is proportional to an identity matrix.

## Appendix F

**Table A6:** ANOVA assumption check for the variable ITPC.

**ROI 1**									
**Homogeneity of Variance ^a^**					**Normality ^b^**				
**Stimulus Type**		**Levene st.**	**df1**	**df2**	***p***	**Group**	**Shap.–Wilk st**	**df**	***p***
D1		2.806	2	44	0.071	Adults	0.953	20	0.407
						TLD	0.967	11	0.86
						DLD	0.891	16	0.059
D2		0.316	2	44	0.731	Adults	0.928	20	0.141
						TLD	0.932	11	0.428
						DLD	0.879	16	**0.037**
D3		2.198	2	44	0.123	Adults	0.965	20	0.64
						TLD	0.928	11	0.39
						DLD	0.931	16	0.25
D4		2.291	2	44	0.113	Adults	0.944	20	0.286
						TLD	0.945	11	0.583
						DLD	0.968	16	0.811
St		2.746	2	44	0.075	Adults	0.872	20	**0.013**
						TLD	0.983	11	0.982
						DLD	0.926	16	0.211
**ROI2**									
**Homogeneity of Variance ^a^**					**Normality ^b^**				
**Stimulus** **Type**		**Levene st.**	**df1**	**df2**	***p***	**Group**	**Shap.–Wilk st**	**df**	***p***
D1		1.748	2	44	0.186	Adults	0.97	20	0.761
						TLD	0.849	11	**0.042**
						DLD	0.981	16	0.97
D2		0.980	2	44	0.383	Adults	0.941	20	0.245
						TLD	0.972	11	0.903
						DLD	0.945	16	0.419
D3		4.083	2	44	**0.024**	Adults	0.961	20	0.555
						TLD	0.915	11	0.278
						DLD	0.956	16	0.592
D4		1.916	2	44	0.159	Adults	0.912	20	0.069
						TLD	0.933	11	0.439
						DLD	0.877	16	**0.035**
St		0.453	2	44	0.639	Adults	0.97	20	0.761
						TLD	0.849	11	**0.042**
						DLD	0.981	16	0.97
**Equality of Covariance Matrices ^c^**					**Sphericity ^d^**				
**ROI**	**Box’s M**	**F**	**df1**	**df2**	***p***	**Mauchly’s W**	**Approx. Chi^2^**	**df**	***p***
1	35.75	0.974	30	3795	0.506	0.779	10.57	9	0.306
2	36.03	0.982	30	3795	0.495	0.845	7.13	9	0.624

Note. Significant tests are indicated in bold fonts. ^a^, ^b^, ^c^, ^d^, as in Appendix C.
